# Conservative Laparoscopic Approach for the Management of a 14-Week Viable Ectopic Cesarean Scar Ectopic Pregnancy

**DOI:** 10.1155/2024/6682029

**Published:** 2024-10-04

**Authors:** Wael Elbanna, Osama Azmy

**Affiliations:** ^1^Obstetrics and Gynaecology, Hayat Woman Center, Cairo, Egypt; ^2^Reproductive Health Department, National Research Center, Cairo, Egypt; ^3^Obstetrics and Gynaecology, Egypt Centre for Research and Regenerative Medicine (ECRRM), Cairo, Egypt

**Keywords:** cesarean scar ectopic, ectopic pregnancy, laparoscopy

## Abstract

**Introduction:** Cesarean scar ectopic pregnancy (CSEP) is a rare gynecological disorder that occurs at a rate of approximately 0.05% of pregnancies and less than 0.2% of cesarean scars. The ultimate goal in the management of CSEP cases is to remove pregnancy and reduce morbidity while preserving fertility. This case report highlights the successful application of a conservative laparoscopic approach in managing a 14-week viable CSEP.

**Case Presentation:** A 35-year-old multiparous woman (G8P5A2L5) with five previous cesarean sections and five normal healthy children presented to the clinic with a viable CSEP of 14 weeks of gestation as revealed by abdominal and transvaginal ultrasound examination. The decision for a conservative laparoscopic approach was made in light of the patient's desire to preserve fertility.

**Intervention and outcome:** The laparoscopic procedure included the following steps: extensive dissection of adhesions between the bladder and the uterus; identification of the ectopic pregnancy at the level of the lower segment; extraction of the product of conception in an endobag; and suturing of the lower segment defect. The successful execution of these steps resulted in the removal of the ectopic pregnancy while addressing associated structural concerns. This approach allowed for mitigating morbidity and, importantly, preserving the patient's fertility.

**Conclusion:** This case highlights the importance of a conservative laparoscopic approach for CSEP in the second trimester. Imaging techniques play a pivotal role in accurate diagnosis, with minimally invasive technologies offering effective solutions. Individualized, patient-centered approaches are necessary to prioritize clinical outcomes and patient preferences.

## 1. Introduction

An ectopic pregnancy occurs when a fertilized egg implants outside the uterus, most commonly in the fallopian tubes, and can occur in other places, such as the cervix, ovaries, or abdominal cavity [1]. It is considered a medical emergency case as the developing embryo can rupture internal organs if left untreated, resulting in severe internal bleeding that could be fatal [2]. Thus, early detection and medical intervention are critical to avoid complications. The most commonly used intervention for ectopic pregnancy is intramuscular injection of methotrexate and surgical removal of the pregnancy [2, 3].

Cesarean scar ectopic pregnancy (CSEP) is a rare type of ectopic pregnancy. However, with the increasing number of cesarean deliveries, the incidence of CSEP is expected to rise. Managing CSEP poses a unique challenge as various surgical approaches are available, such as transvaginal surgery, laparoscopy, laparotomy, or curettage. Additionally, alternative treatment modalities such as uterine artery embolization (UAE), administration of methotrexate, potassium chloride injection, needle-guided sac decompression, high-intensity focused ultrasound imaging, balloon catheters, and combinations of these methods have also been described [2–4].

In this case report, we present a conservative laparoscopic approach for the management of a 14-week viable ectopic cesarean section scar pregnancy. We describe the methodology employed for diagnosis and treatment, highlighting the intricacies of the surgical procedure and the postoperative monitoring of the patient.

## 2. Case Presentation

A 35-year-old multiparous woman with a history of 5 previous cesarean sections resulting in five living, normal children (G8P5A2L5) was initially misdiagnosed with a cervical ectopic pregnancy at different centers and received several courses of methotrexate without response. Upon further examination at our clinic, a color Doppler ultrasound revealed a viable 14-week exophytic CSEP, necessitating an alternative approach to management.

**Figure 1:**
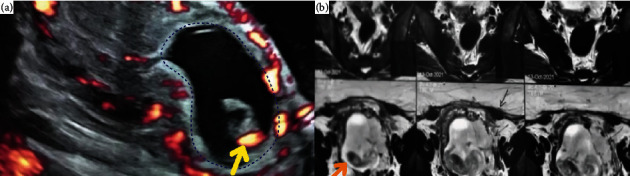
(a) Ultrasound image showing a 14-week cesarean scar ectopic pregnancy, featuring a protruding mass with a regular gestational sac (blue arrow) and viable embryo (yellow arrow), was found at the precise location of the previous C-section scar in the anterior uterine niche. (b) Magnetic resonance imaging illustrating a 14-week cesarean scar ectopic pregnancy, showing intrauterine gestational sac with lower segment bulge, myometrial thinning at the cesarean scar site, intact serosa, and adhesions to the urinary bladder.

Upon physical examination, the patient's blood pressure was 100/60 mmHg, and her pulse rate was 90 beats per minute. Laboratory tests revealed a hemoglobin level of 10 g/dL. An abdominal ultrasound examination revealed a viable ectopic cesarean section scar pregnancy of 14 weeks, as shown in Figure 1(a).

Magnetic resonance imaging was conducted to assess the extent of left lateral invasion of the myometrium. The MRI showed well defined intrauterine gestational sac distending the endometrial cavity located mainly at the lower segment, producing an outward bulge at the site of the cesarean section scar causing myometrial thinning reaching up to 2 mm yet intact overlying serosa, but adhesions between the uterus and urinary bladder are present (Figure 1(b)).

A comprehensive investigation was conducted to ensure the patient's suitability for surgery. This included a series of blood tests such as a complete blood count, liver and kidney function tests, coagulation profile, determination of blood group, assessment of hepatic viral markers, as well as measurements of random blood glucose and HbA1c levels. Furthermore, preoperative assessments encompassed an ECG, echo, and Doppler examinations on both lower limbs (venous and arterial). The patient's blood group was identified, a wide-bore cannula was inserted, and blood was made available as a precautionary measure for any potential incidents.

The management of this case involved a careful consideration of various factors based on the available evidence and the patient's preferences. The decision to pursue the laparoscopic approach was optimal in this scenario as systemic methotrexate administration via intramuscular injection has already been given and failed. Also, methotrexate is typically the medication of choice for medical management when the pregnancy is less than 8 weeks gestation [5]. Intragestational sac methotrexate and hysteroscopic route were avoided for potential complications, such as sac perforation and excessive intraabdominal bleeding, as the patient's pregnancy was at 14 weeks of gestation with an exophytic nature sac. Additionally, the complete extraction of a large-sized baby from the cervix may be associated with a higher failure rate and a greater risk of complications.

The laparoscopic approach was the optimal management strategy in this case. Minimally invasive techniques have been shown to have lower complication rates and higher success rates while also avoiding certain surgical risks, such as adhesions and infections. Patient selection is a crucial factor for achieving optimal results with this approach.

The patient received counseling regarding the risk of future ectopic pregnancies and the potential benefits of tubal ligation. Despite our offer of tubal ligation, she declined vehemently due to cultural and religious beliefs opposing the procedure and her desire to maintain fertility. The patient made an informed decision to undergo operative resection via laparoscopy, prioritizing a faster recovery and a prompt return to work, as well as preserving fertility. This decision-making process, taking into account the patient's preferences and the available evidence, led to the selection of the laparoscopic management approach as the most appropriate choice for this case.

**Figure 2:**
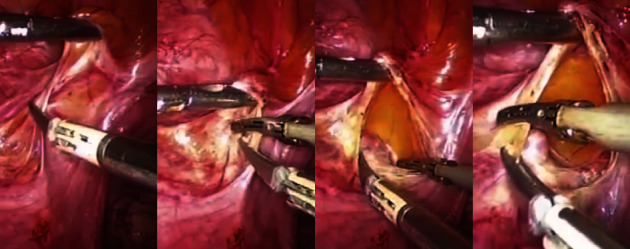
Close-up of laparoscopic operation of a cesarean scar ectopic pregnancy: the initiation of lysis of the adhesions using an ultrasonic scalpel at the level of the lower uterine segment.

Initially, after the introduction of the trocar, the examination of the uterus revealed extensive adhesion to the bladder with obvious varicosities. So, dissection and lysis of adhesions were initiated using an ultrasonic scalpel and bipolar devices at the level of the lower uterine segment (Figure 2). The surgical team proceeded with careful dissection of the bladder until exposing the vaginal area, ultimately identifying the scar pregnancy situated at the lower uterine segment. In order to decrease bleeding, a solution of diluted vasopressin and oxytocin was injected into the myometrium surrounding the gestational sac. Then, the hysterotomy was performed at the site of the identified scar pregnancy.

**Figure 3:**
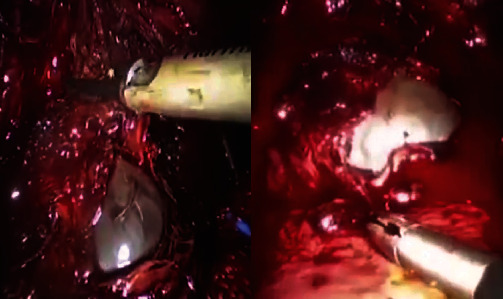
Close-up of laparoscopic operation of a cesarean scar ectopic pregnancy: the identification of the gestational sac.

**Figure 4:**
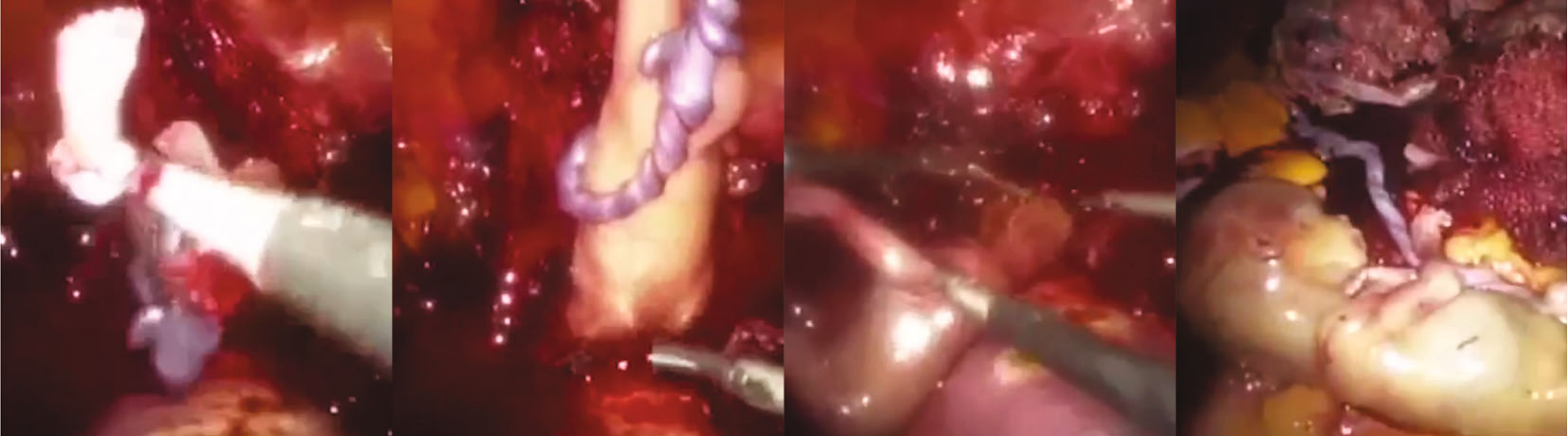
Close-up of laparoscopic operation of a cesarean scar ectopic pregnancy: the extraction of the fetus and placenta.

**Figure 5:**
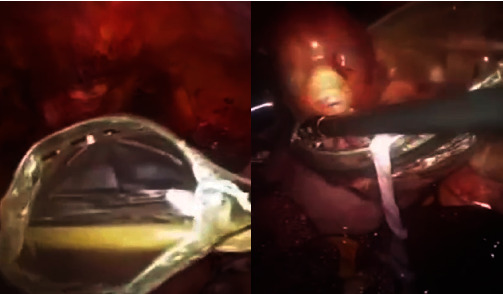
Close-up of laparoscopic operation of a cesarean scar ectopic pregnancy: the use of an endobag to remove the fetus and the placenta.

The gestational sac was successfully identified and extracted (Figure 3). Subsequently, extraction of the fetus and placenta was done (Figure 4), and the use of an endobag facilitated the removal of the fetus and the placenta (Figure 5). The hysterotomy was thoroughly repaired, ensuring adequate hemostasis, using a continuous barbed suture technique in two layers.

Postoperatively, the patient was closely monitored for vital parameters and was discharged after 24 h. Serial testing revealed a normalization of beta-HCG levels within a period of 2 weeks following the operation.

## 3. Discussion

In recent years, the incidence of CSEP cases has increased, attributed to the rise in cesarean deliveries [6]. However, the available literature and data available specifically on CSEP cases are limited, particularly for those that progress to the second trimester. To date, the etiology and pathophysiology of the CSEP are not fully understood [7].

Currently, there is no established gold standard for managing CSEP. Expectant management yields a poor prognosis with a high risk of maternal complications, potentially necessitating an emergency hysterectomy [8, 9]. Previous management approaches have included systemic and local administration of methotrexate, local injection of potassium chloride (KCl), hysterectomy, dilatation and curettage (D&C), open or laparoscopic scar resection, aspiration, embolization, and hysteroscopy [2, 3, 6, 9]. However, current research does not support using methotrexate alone [10]; in the most recent comprehensive and systematic evidence, systemic methotrexate was reported for 339 women in 3 RCTs and 18 case series, showing that additional treatment was necessary in one-fourth of the cases, and severe complications were seen in 13% [3].

Recent studies support the use of laparoscopy as a minimally invasive procedure [7, 11]. While management strategies for CSEP are usually reported for the first trimester and have been relatively straightforward due to the smaller size of the gestational sac and fetus, their effectiveness and feasibility in later stages of gestation remain largely unexplored.

Nevertheless, the successful application of the laparoscopic approach in managing a 14-week viable cesarean section scar ectopic pregnancy highlights its potential applicability in similar cases. The combined use of transvaginal ultrasound (TVUS) and MRI serves as a valuable diagnostic and surgical planning approach, ensuring a thorough evaluation and facilitating appropriate management for this complex condition. The diagnosis of CSEP was established using TVUS, a readily available imaging technique. The TVUS revealed a well-encapsulated, protruding mass situated in the anterior uterine isthmus, precisely at the site of the previous C-section scar. This mass exhibited a regular gestational sac with a viable single embryo. These findings aligned with the criteria described in previous case reports and clinical guidelines for diagnosing CSEP [7, 12].

The TVUS provided sufficient information on the location, size, and viability of the pregnancy, allowing for prompt and appropriate medical management. However, the MRI was important before planning the surgery, as it provided detailed information regarding the depth of myometrial invasion, the potential involvement of adjacent structures such as the bladder, and overall anatomical considerations.

The laparoscopic procedure employed in this case necessitated meticulous dissection and lysis of adhesions. The patient's history of five cesarean sections resulted in extensive adhesions, posing challenges during the dissection process. The presence of a delicate boundary between the bladder and the ectopic pregnancy further complicated the dissection process.

Remarkably, despite the anticipated substantial bleeding during the hysterotomy, only 160 cc of blood was lost, which is unexpected considering the gestational age of 14 weeks. This outcome is likely attributable to the high pressure of the pneumoperitoneum and the prompt repair of the hysterotomy in addition to the administration of oxytocin and vasopressin. Furthermore, the multiple doses of methotrexate administered prior to surgery may have reduced bleeding during the procedure.

The conservative laparoscopic approach not only avoided the complications and extended hospitalization associated with laparotomy but also effectively addressed the presence of a cesarean scar niche, potentially reducing the risk of recurrence.

## 4. Conclusion

In conclusion, our case report highlights the successful implementation of conservative management in a CSEP case of advanced gestation and a large-sized baby using the expertise of an experienced and skilled laparoscopic surgeon in conjunction with a comprehensive expert medical team. To understand and improve patient outcomes, further research is imperative, focusing on evaluating minimally invasive technologies and their long-term efficacy, particularly regarding recurrence rates. Additionally, exploring the potential benefits of this approach in milder CSEP cases and other relevant clinical scenarios would provide valuable insights for optimizing management strategies. By continuously investigating these areas, we can enhance patient care and refine treatment approaches for CSEP. Exploring the laparoscopy approach further could be a valuable research area in our region, particularly for patients who decline tubal ligation after multiple cesarean sections due to cultural and religious beliefs.

## Data Availability

The patient-level data of this case report is not publicly available due to privacy concerns. However, data can be made available upon reasonable request from the corresponding.
