# Supplementary material for: Conservative Laparoscopic Approach for the Management of a 14-Week Viable Ectopic Cesarean Scar Ectopic Pregnancy
Source: Case Rep Obstet Gynecol. 2024 Oct 4;2024:6682029. doi: 10.1155/2024/6682029 (PMC11469927; doi:10.1155/2024/6682029)
Supplement: Supporting Information — Additional supporting information can be found online in the Supporting Information section. Supporting information further illustrates our case with the laparoscopic procedure. [file 6682029.f1.pdf]

Laparoscopic management of a 14-weeks viable ectopic cesarean section Scar pregnancy

Dr wael elbanna
OB.GYN Consultant
European board of obstetrics and gynecology
M.B.B.CH., M.SC., EBCOG-EFOG.

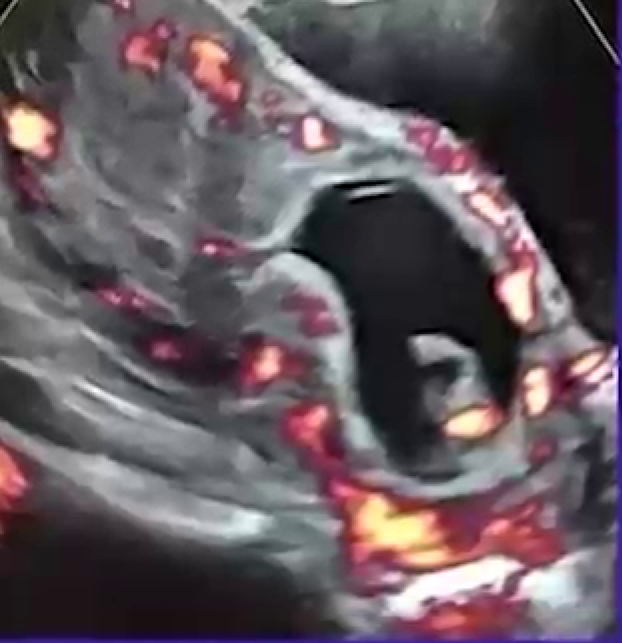

Case presentation

CSEP

A 35-year-old multiparous woman (G8P5A2) presented to the clinic with a diagnosis of cervical ectopic pregnancy. She had received a methotrexate course, . However, abdominal and transvaginal ultrasound examination revealed a viable cesarean section ectopic pregnancy of 14 weeks of gestation. Magnetic resonance imaging was conducted to assess the extent of left lateral invasion of the myometrium. The patient was informed about the treatment options available and opted for conservativ[illegible] management through laparoscopy.

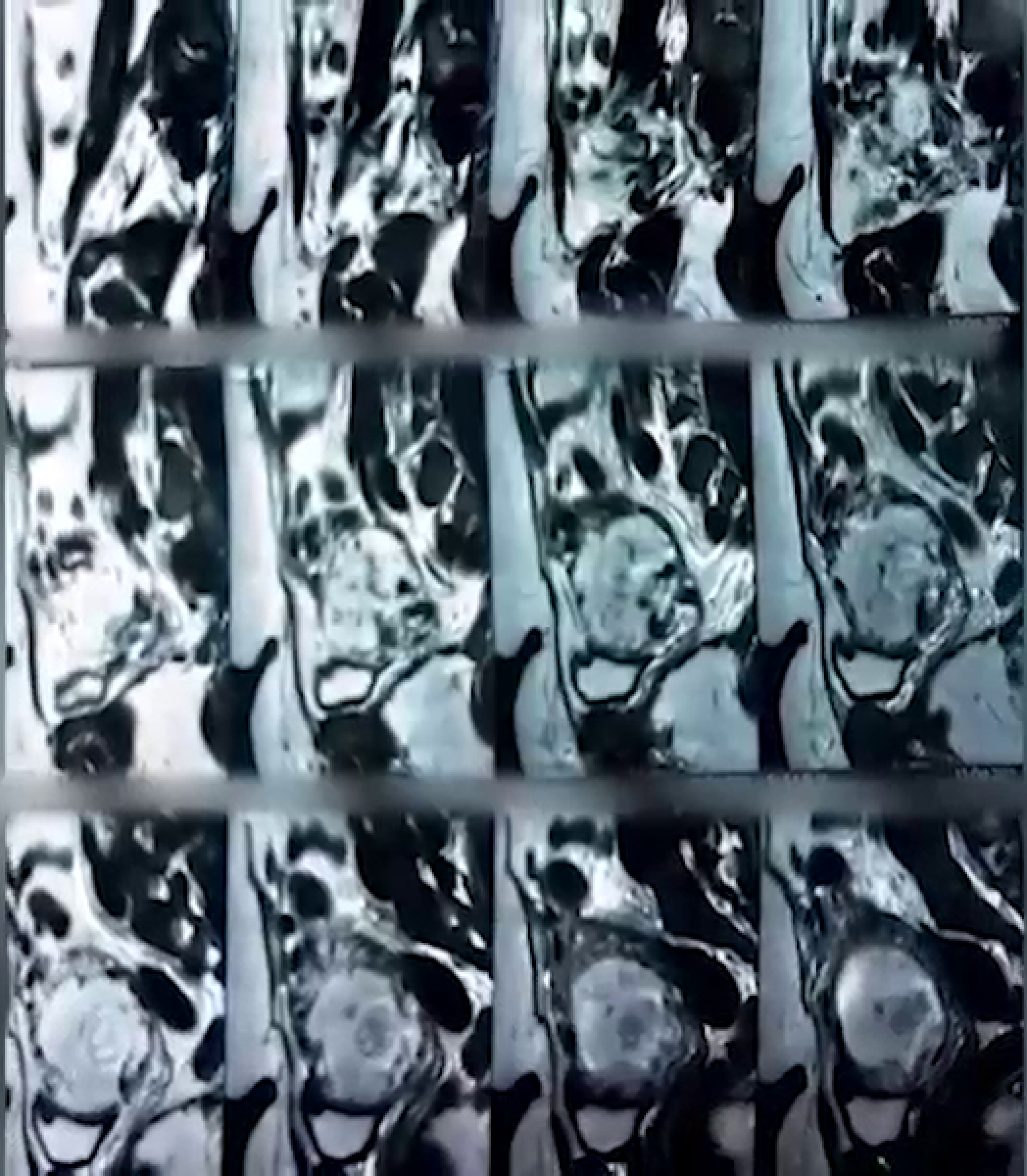

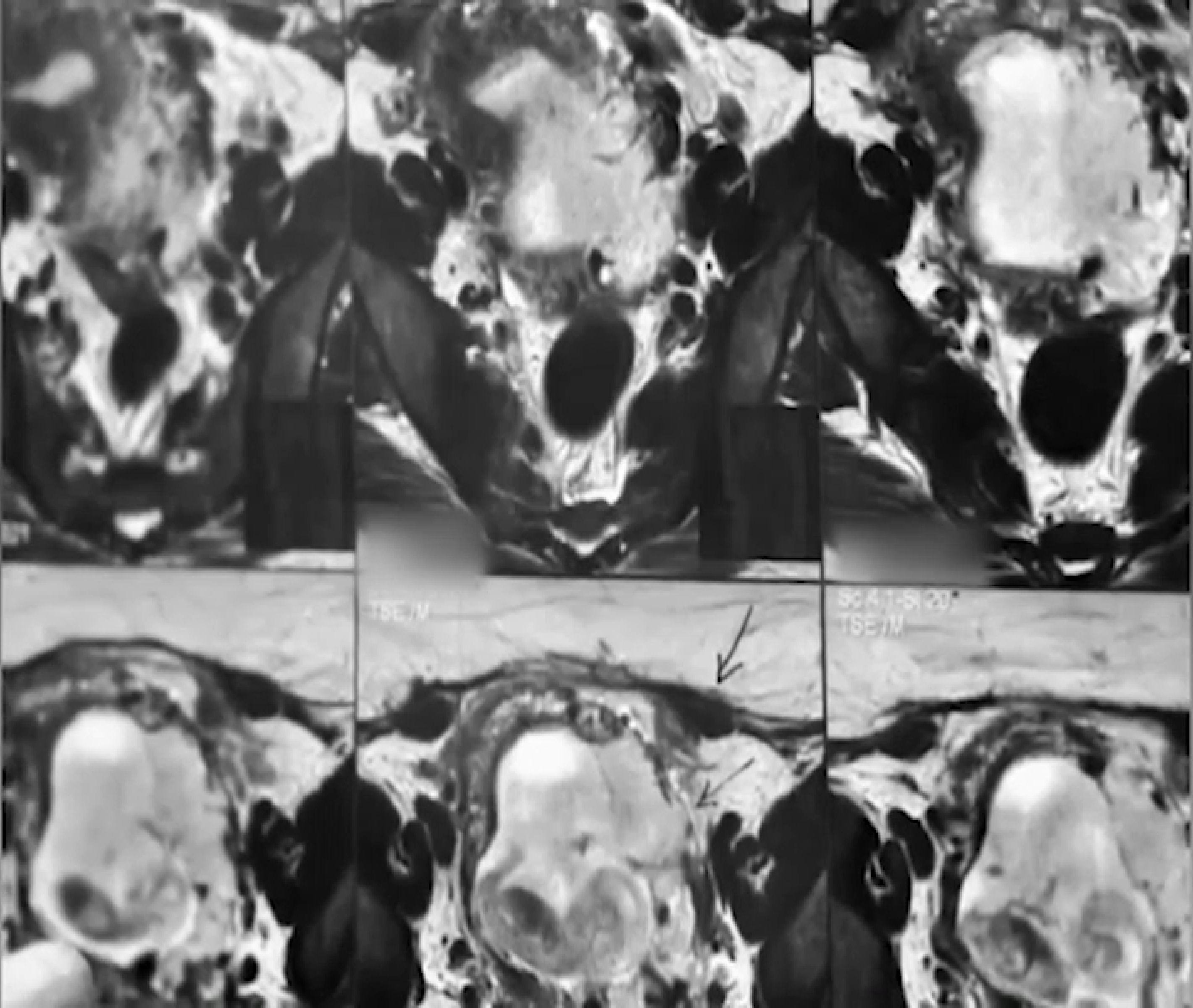

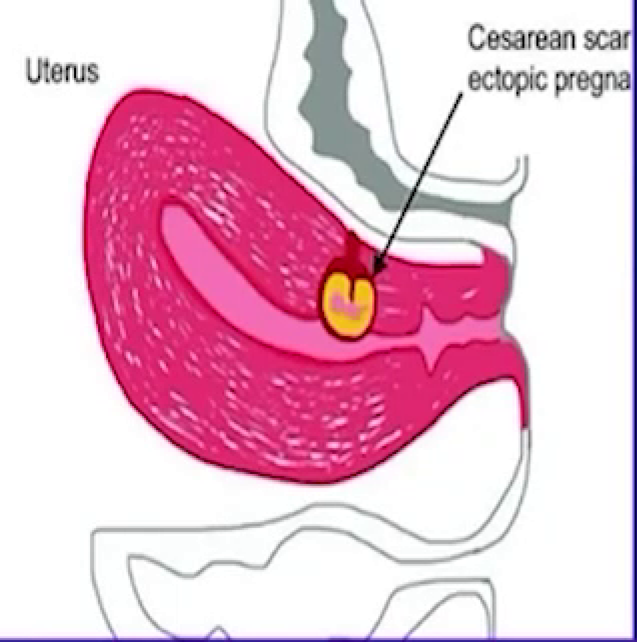

Case presentation

CSEP

Surgical Approach

1. Thorough evaluation for pregnancy location using laparoscopy and MRI
2. Incorporation of intraoperative ultrasound
3. Hemostatic measures
4. Complete enucleation of products of conception
5. Closure of myometrial defect

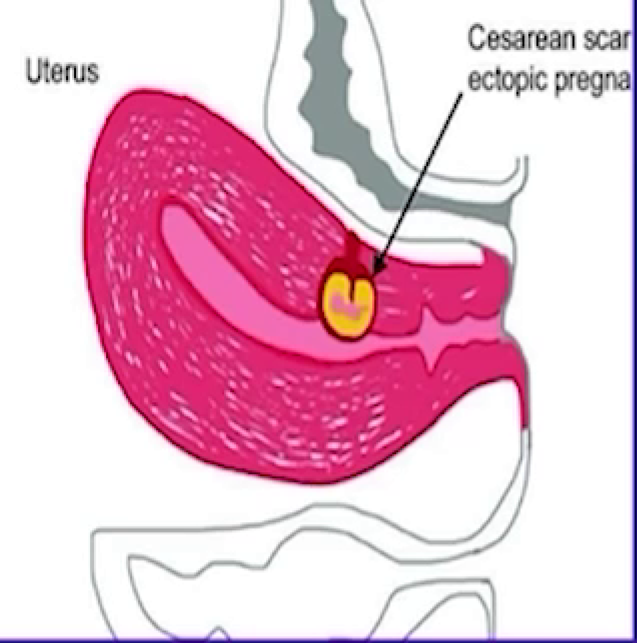

Case presentation

CSEP

Surgical Approach

1. Thorough evaluation for pregnancy location using laparoscopy and MRI
2. Incorporation of intraoperative ultrasound
3. Hemostatic measures
4. Complete enucleation of products of conception
5. Closure of myometrial defect

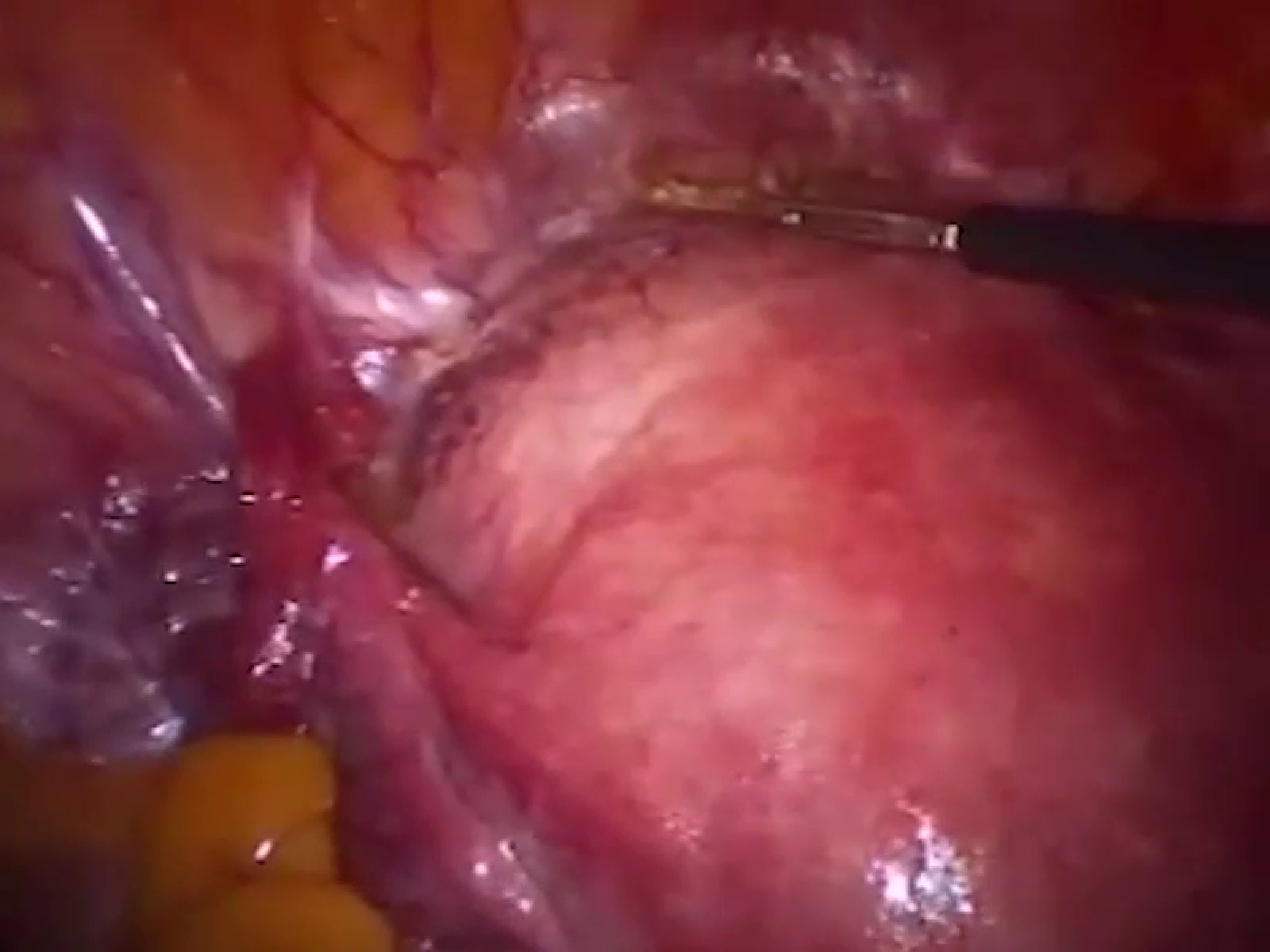

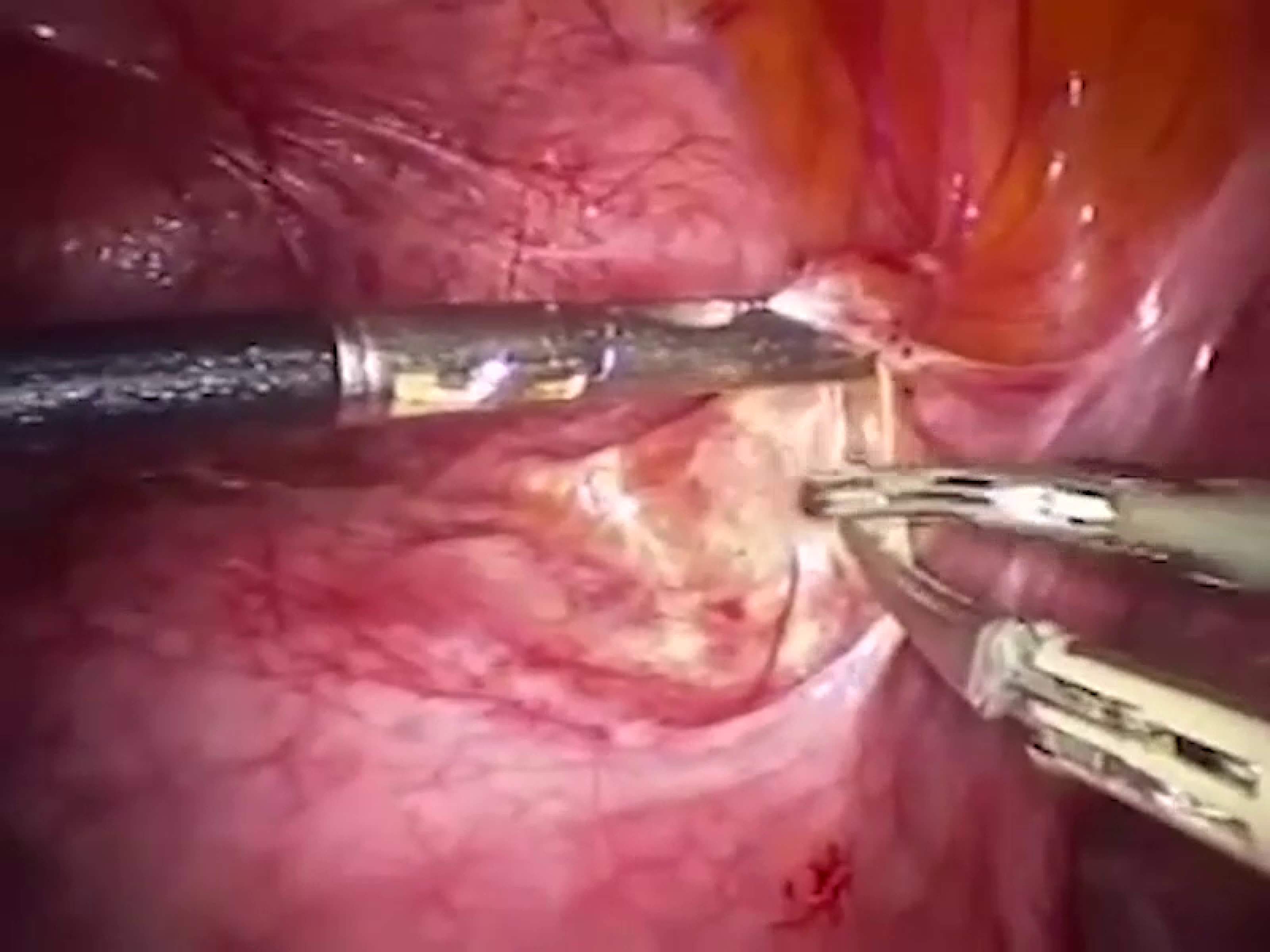

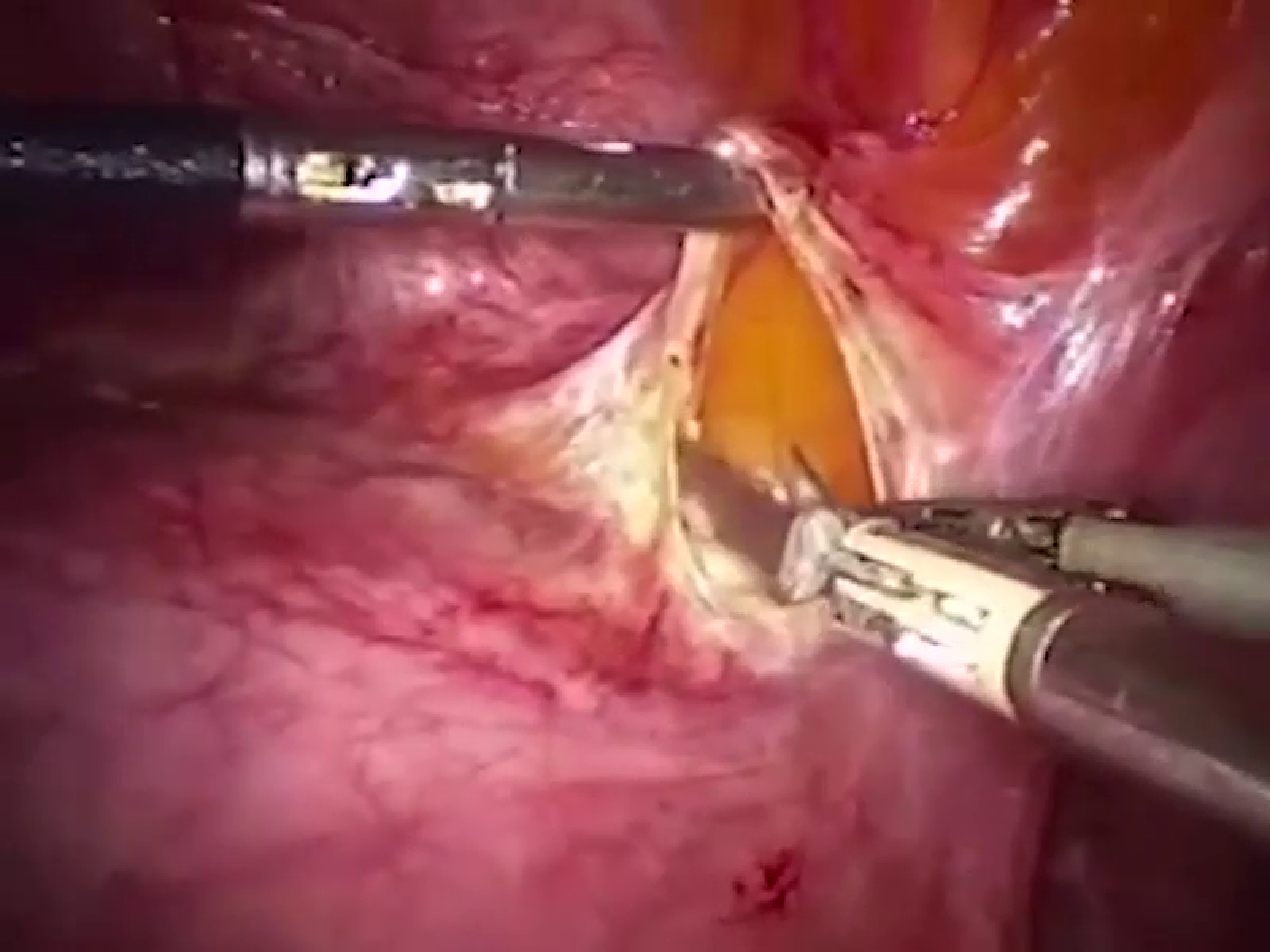

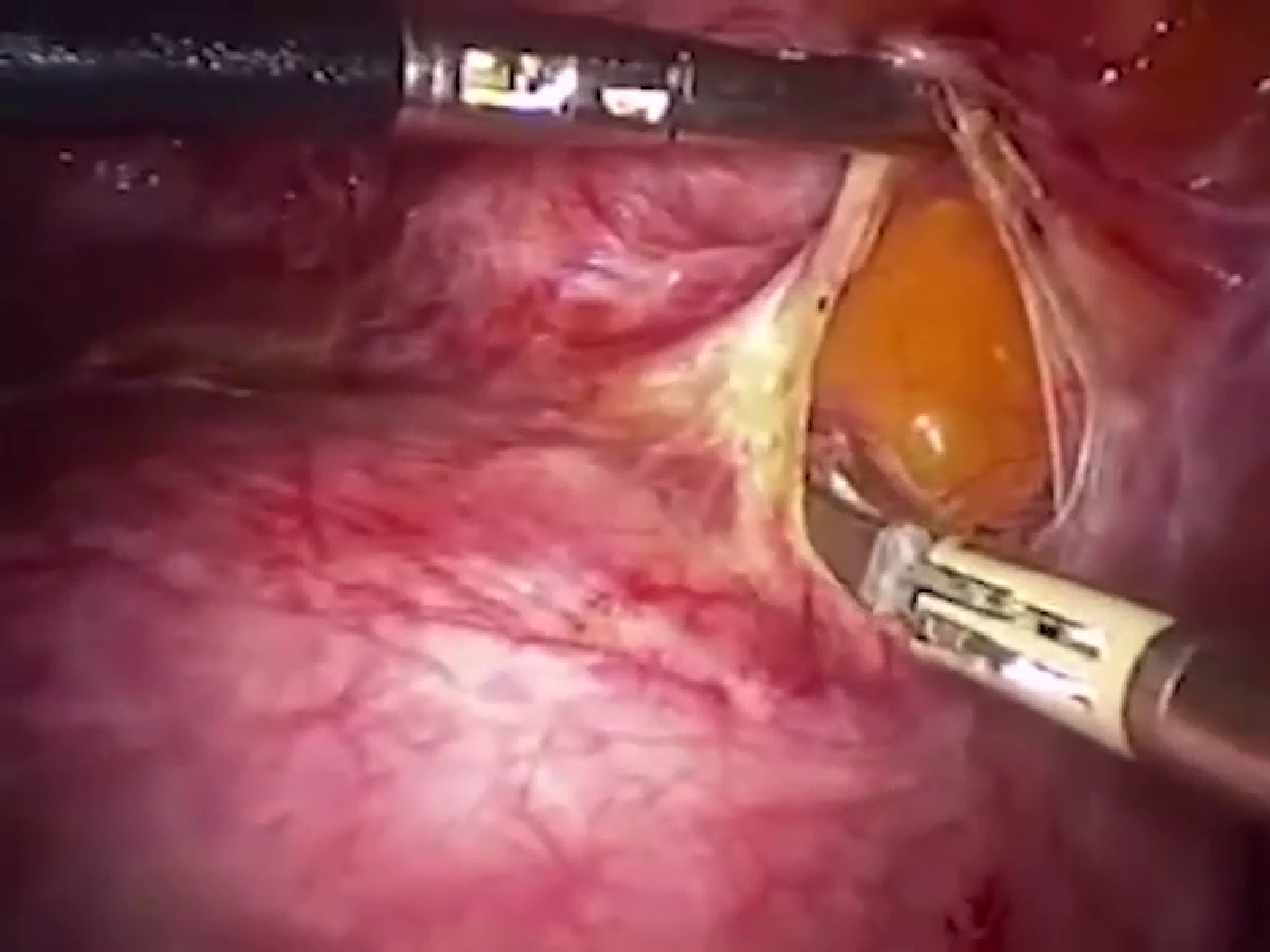

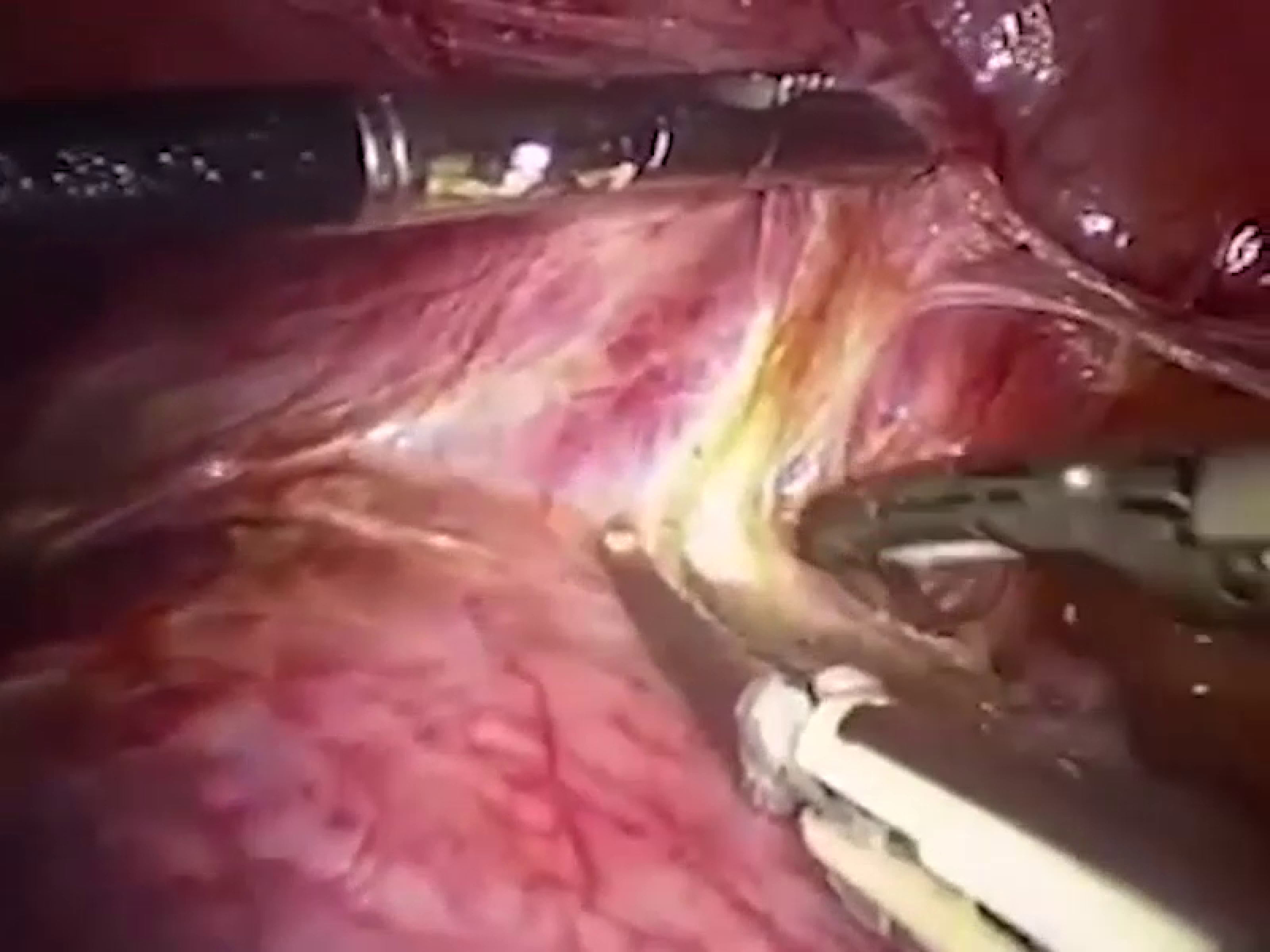

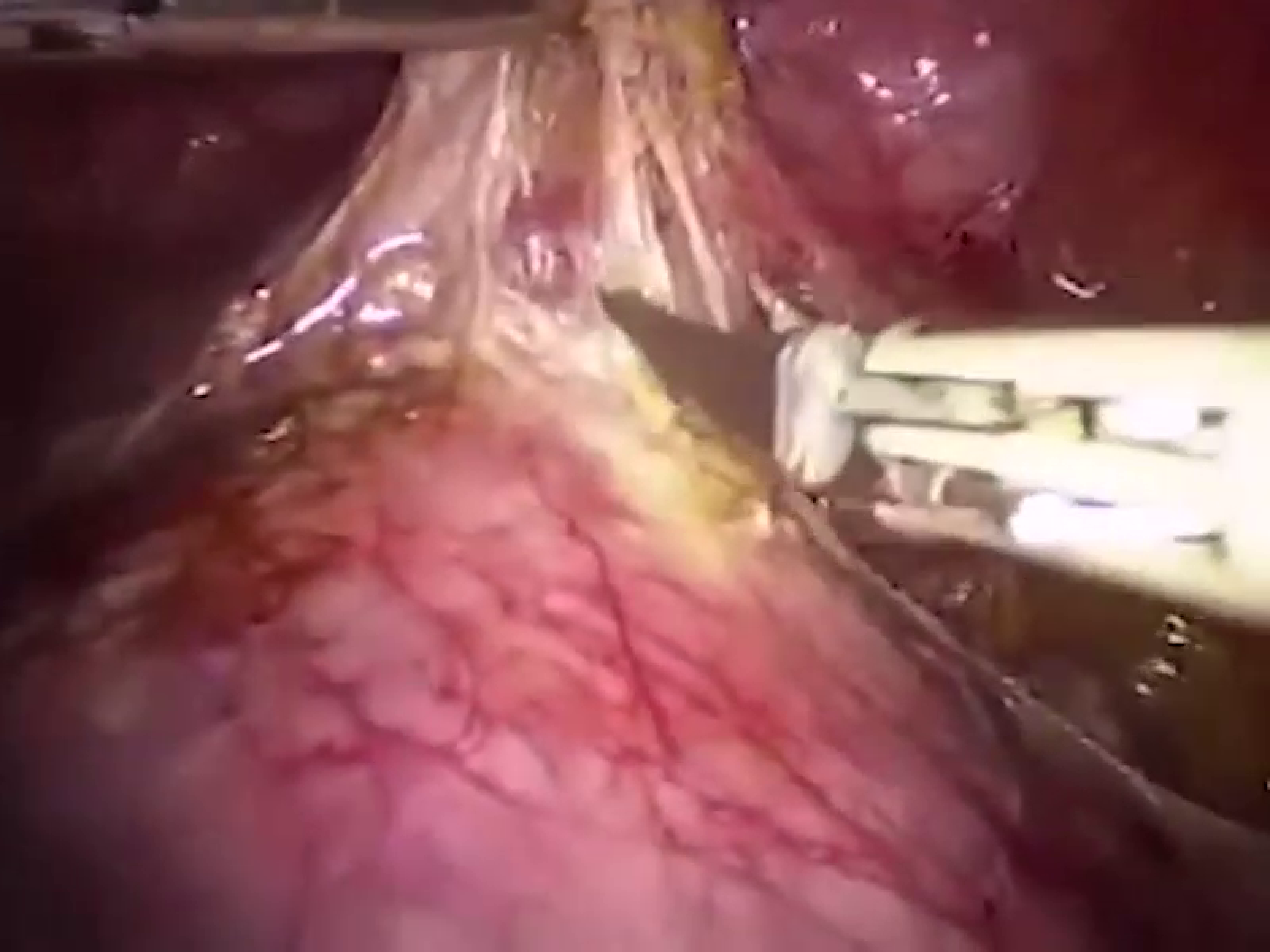

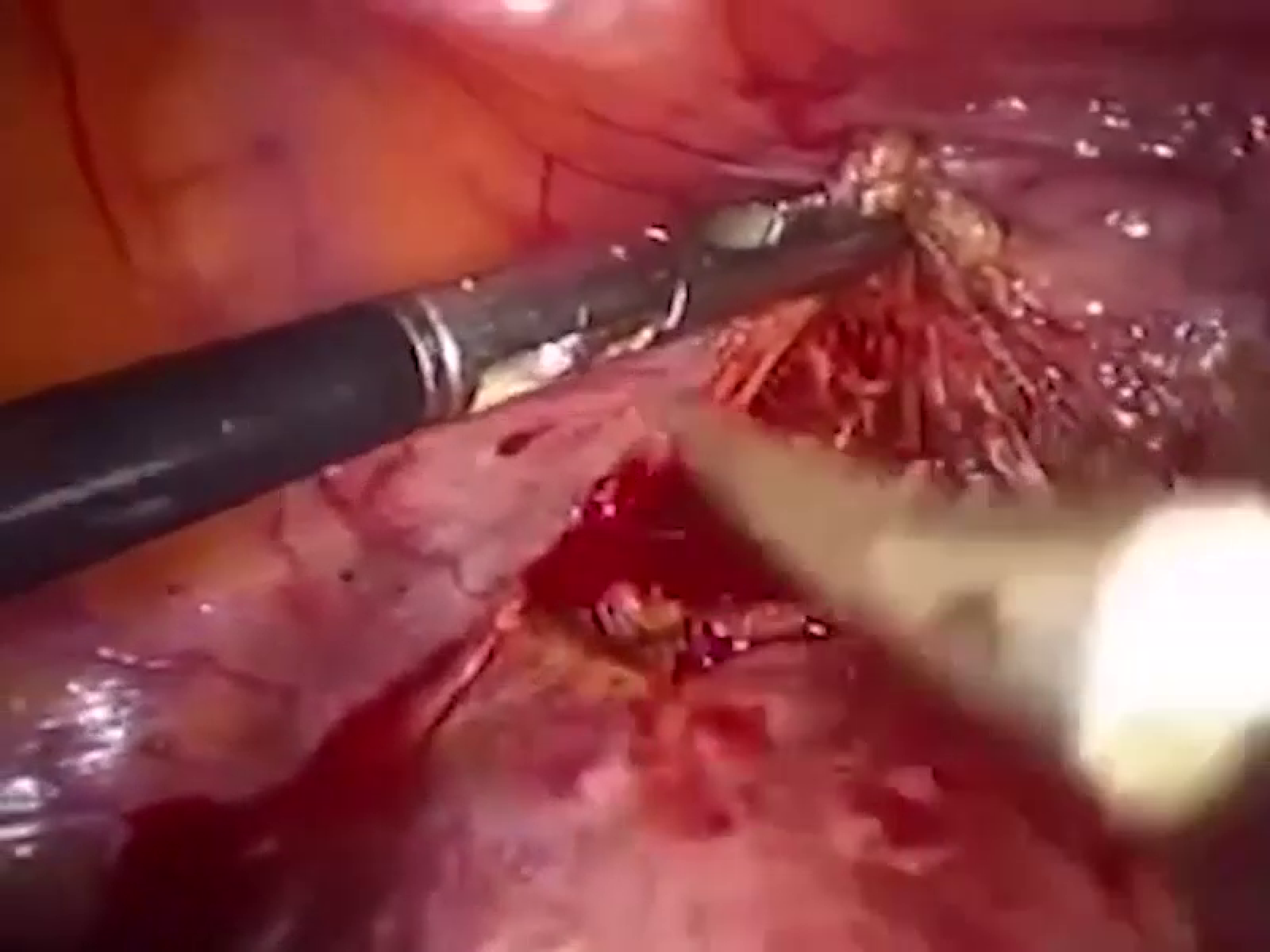

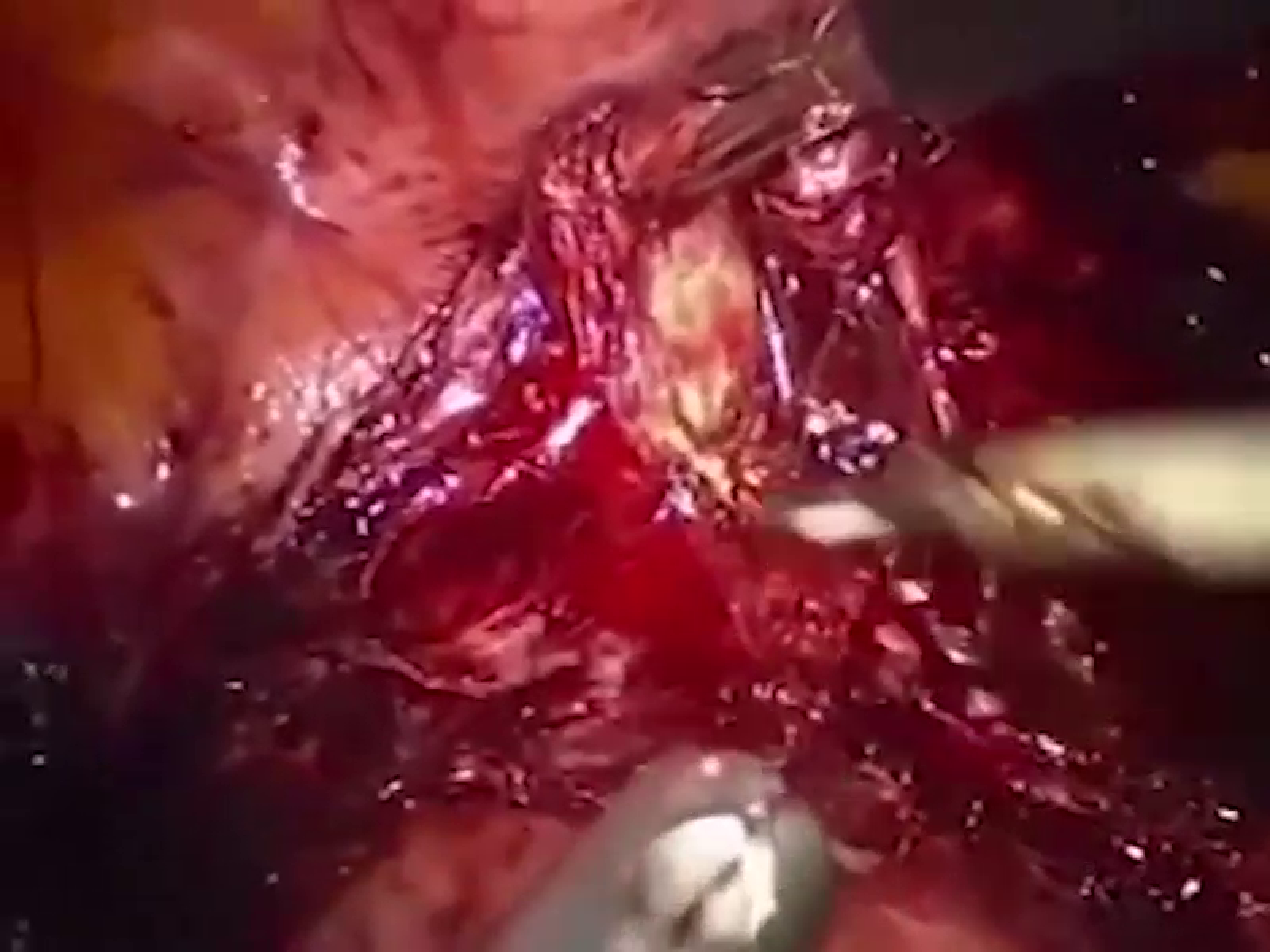

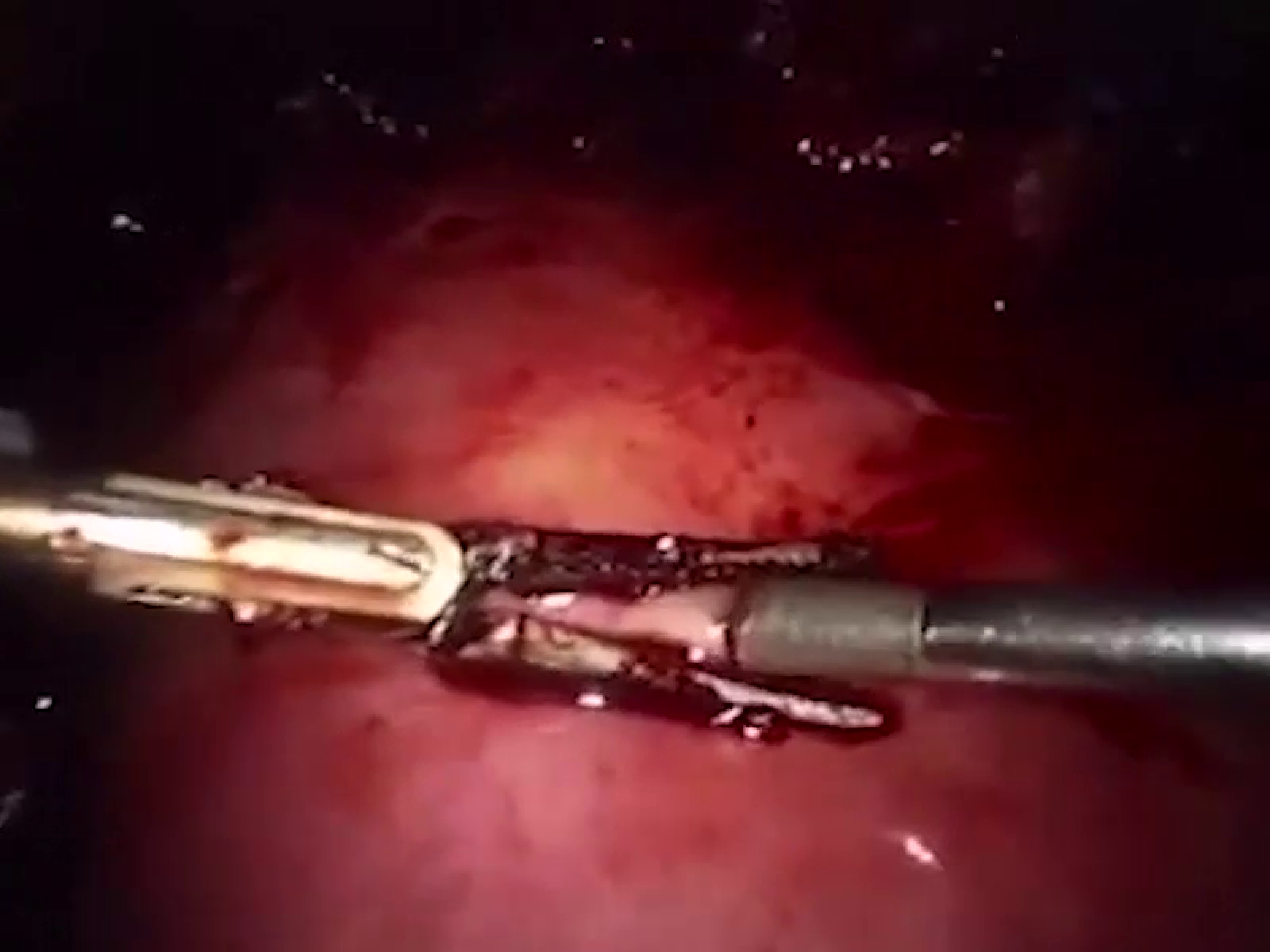

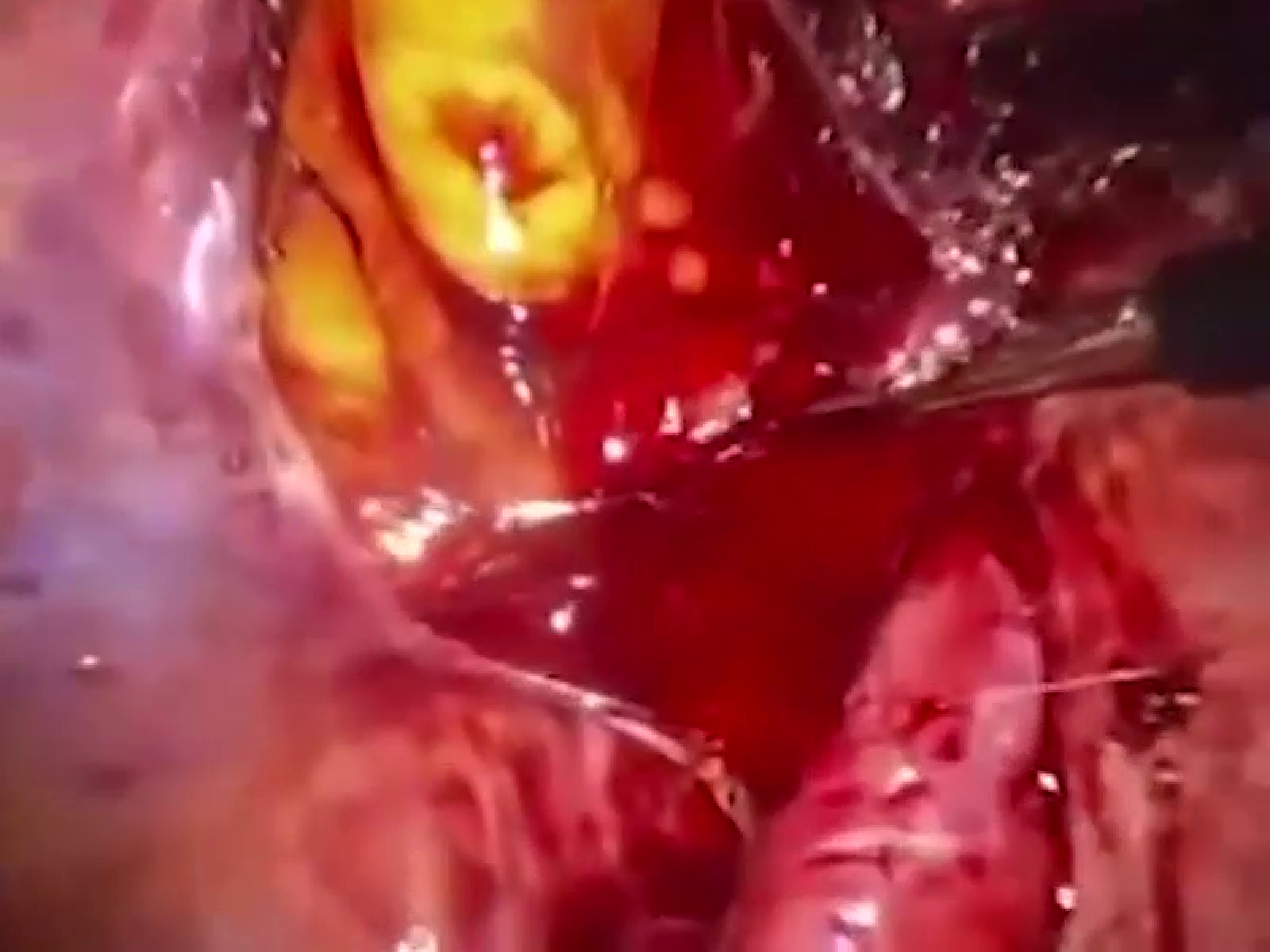

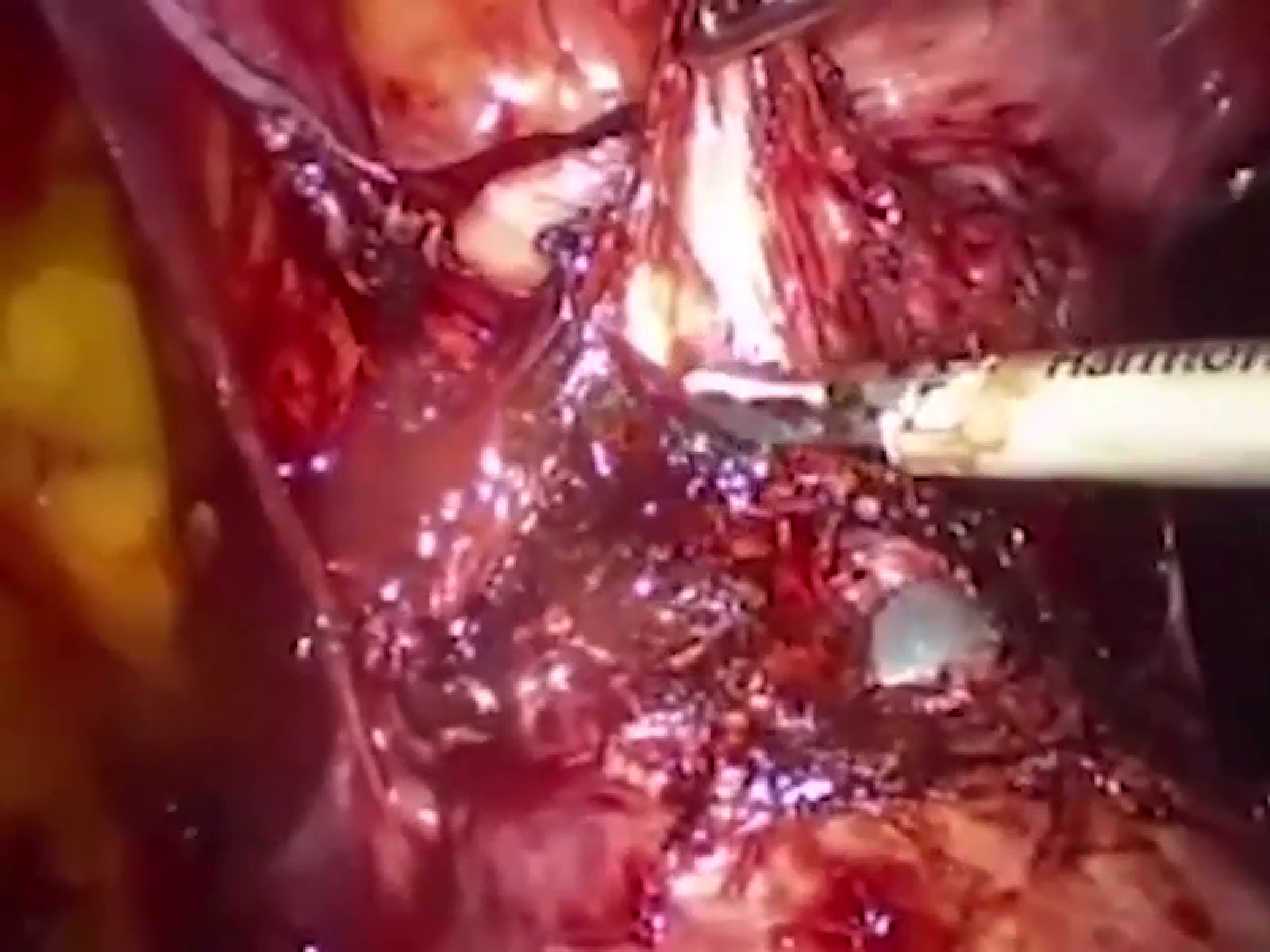

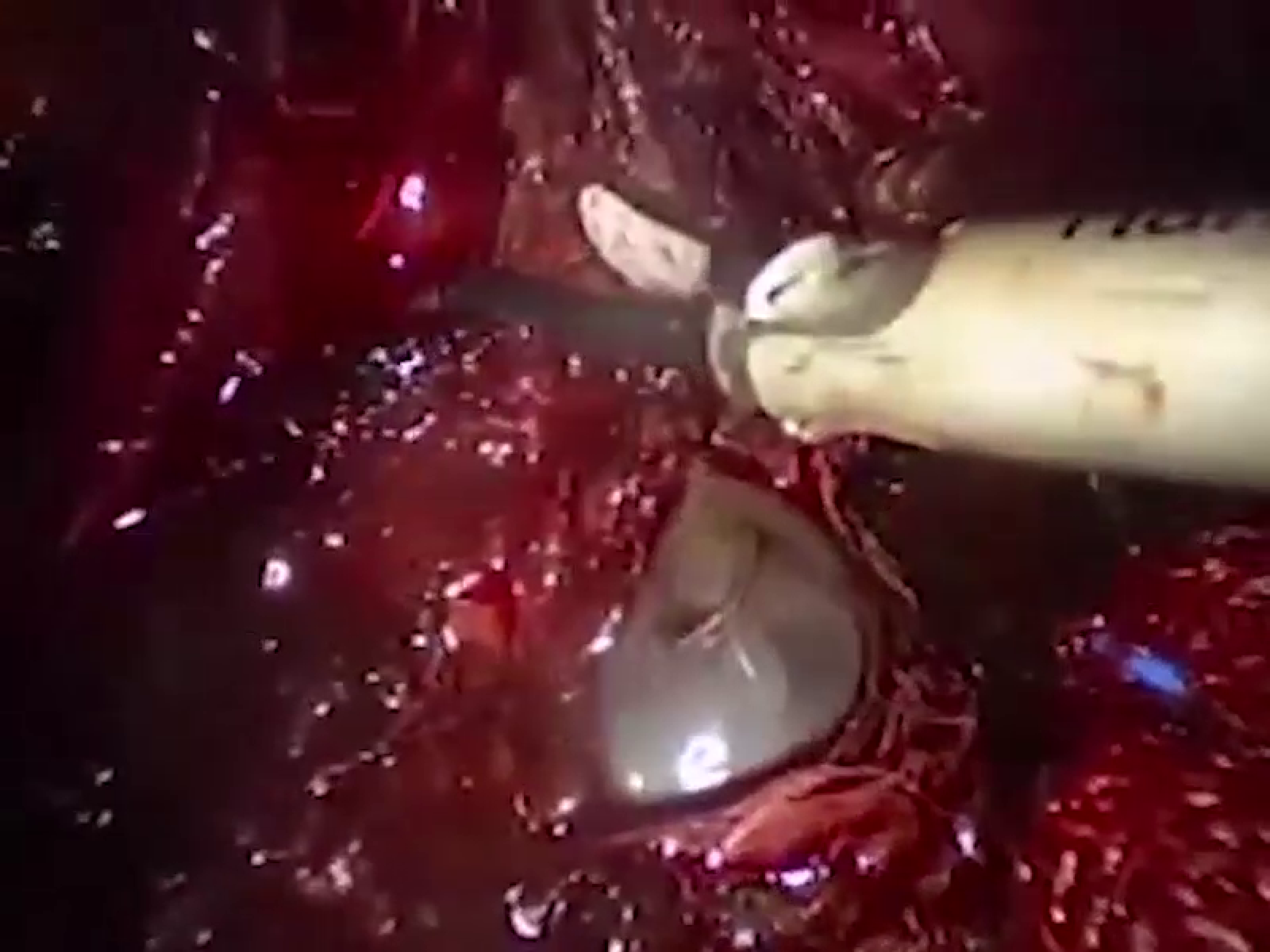

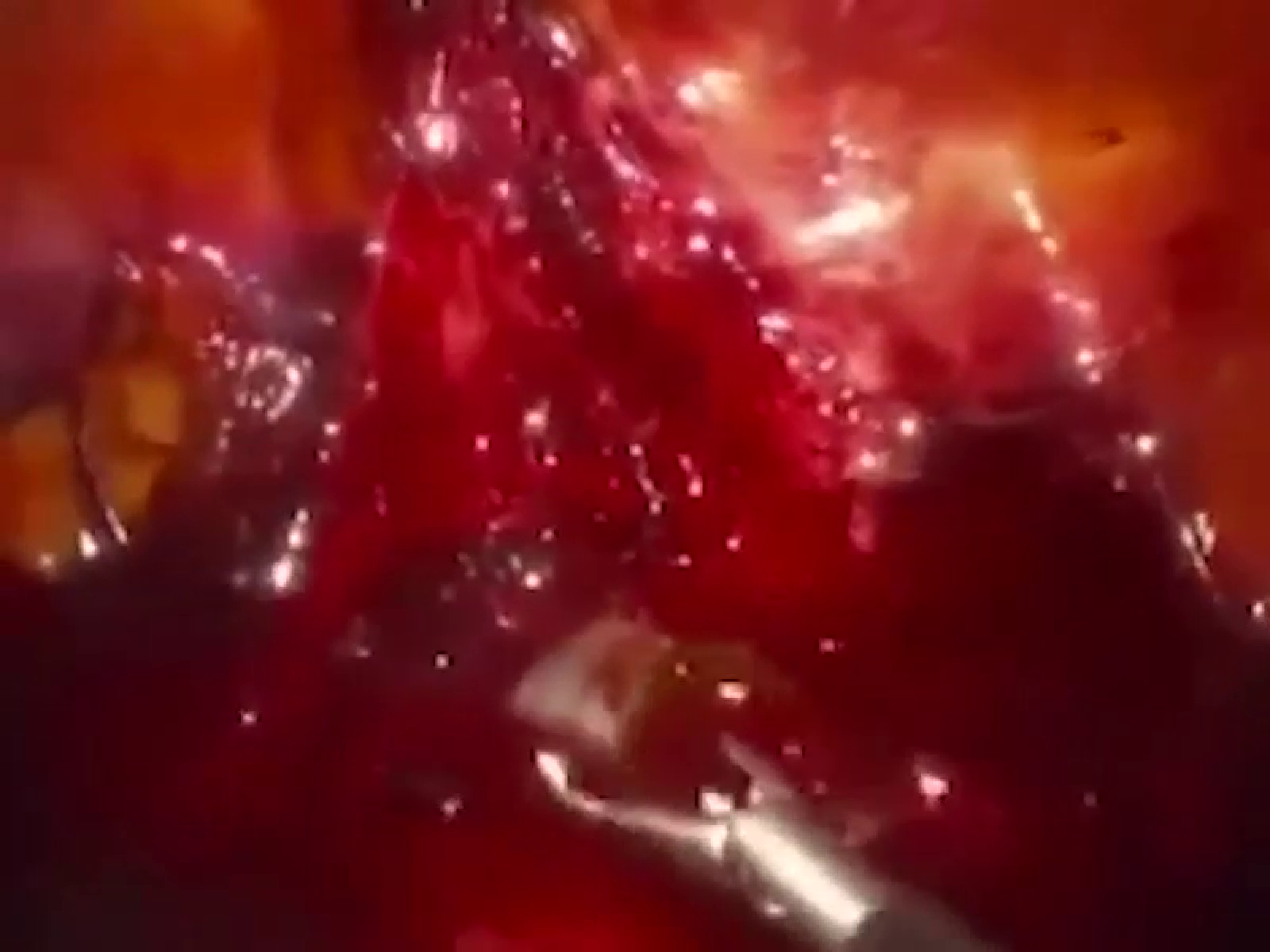

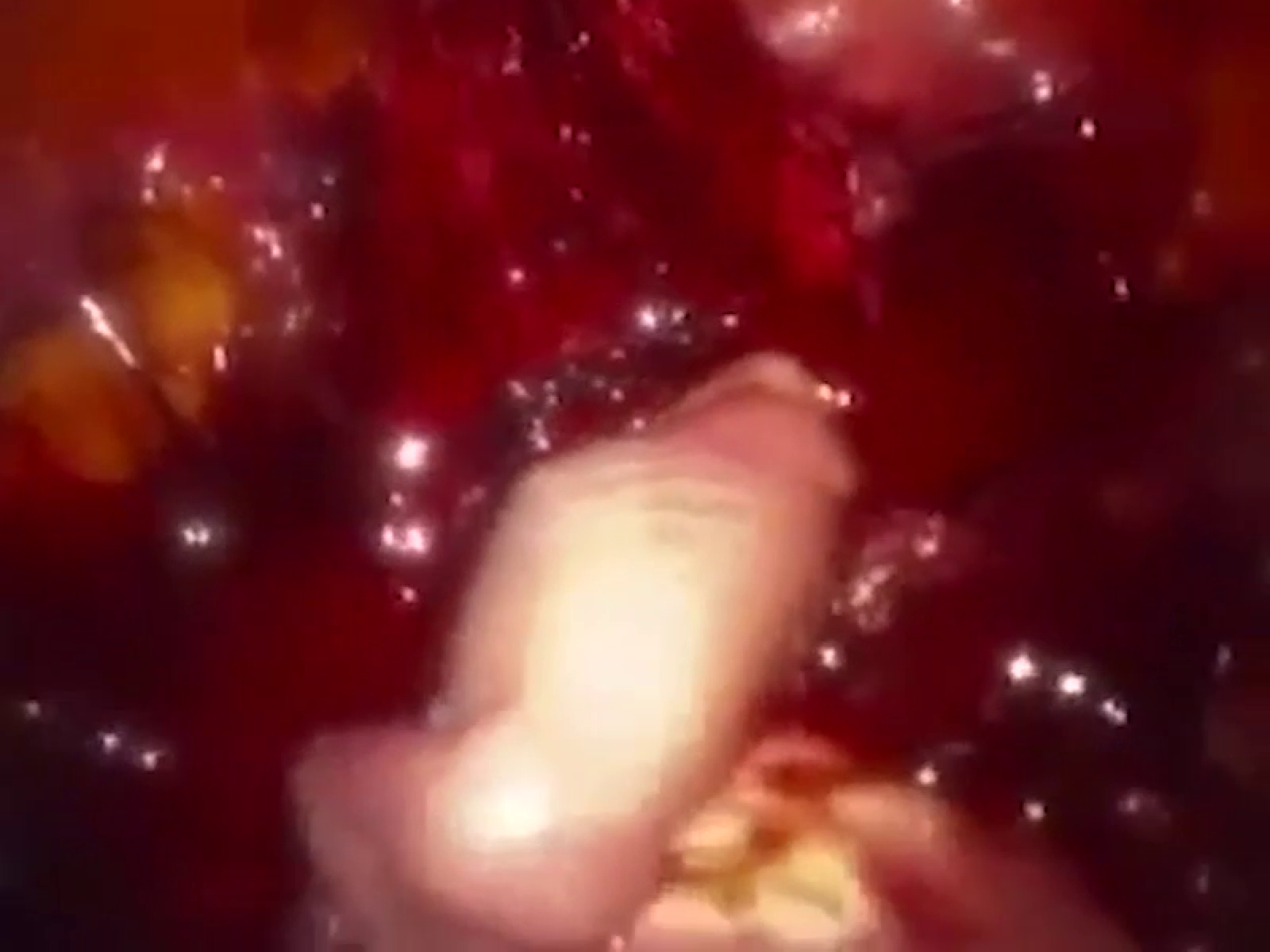

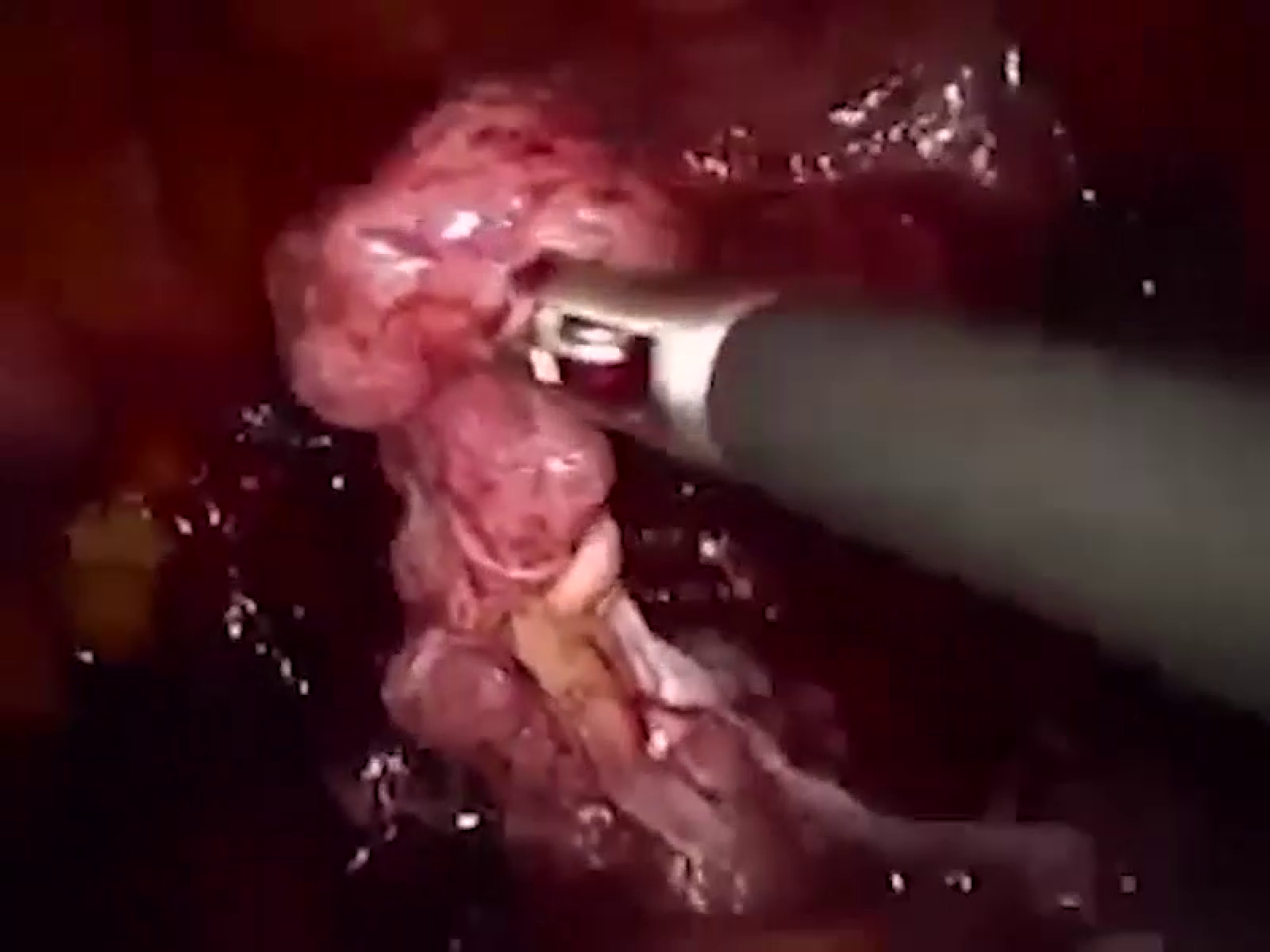

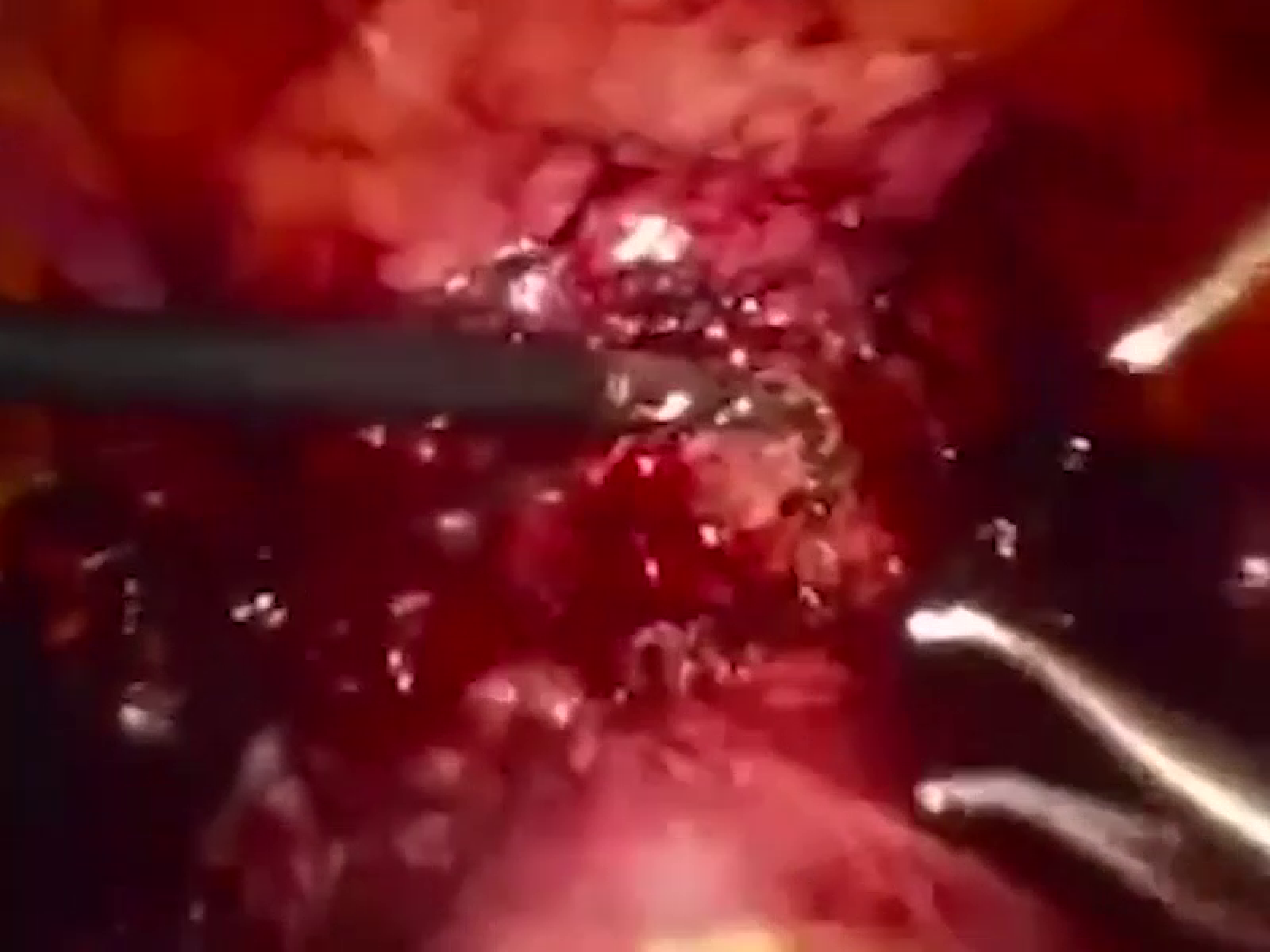

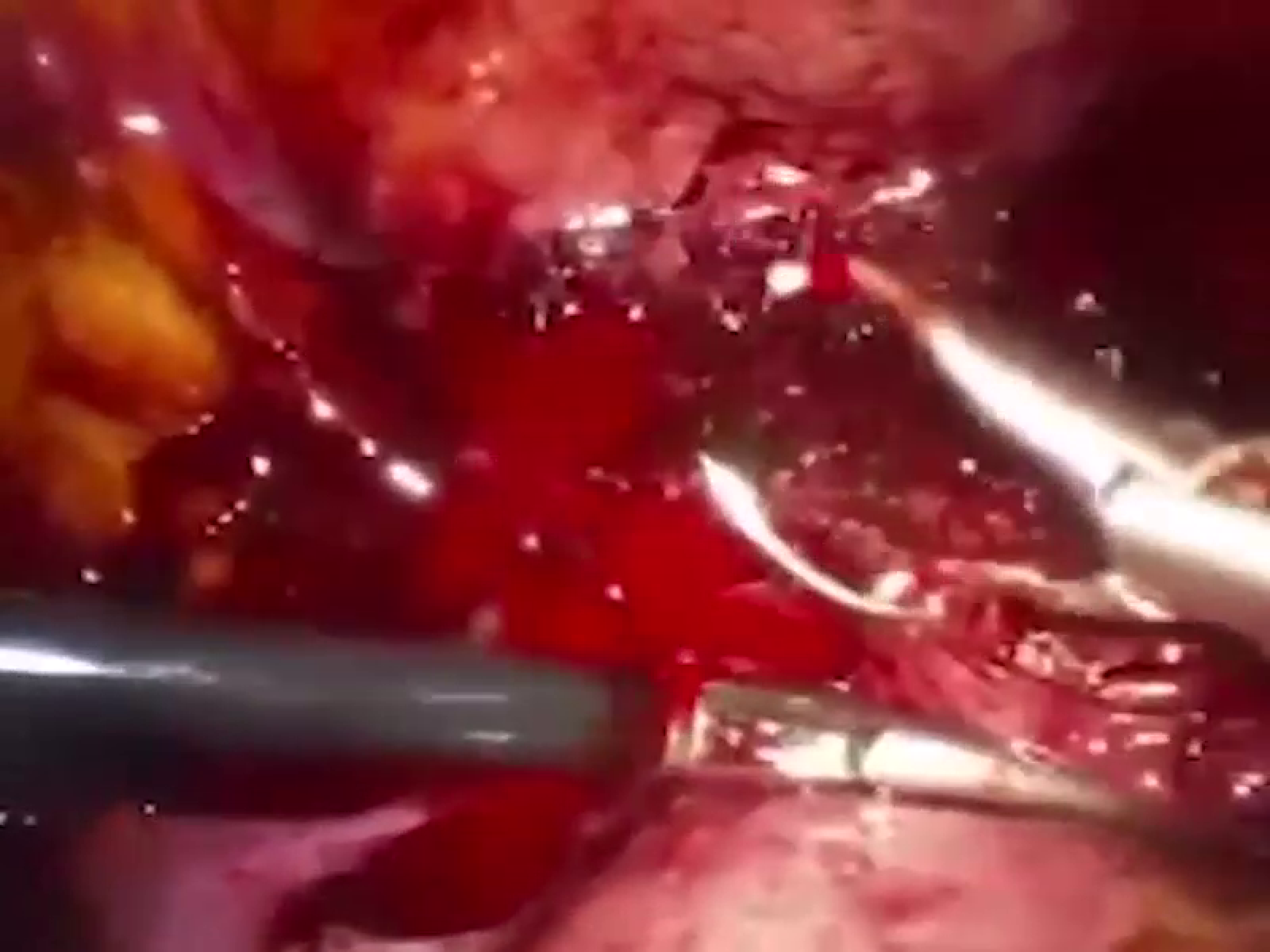

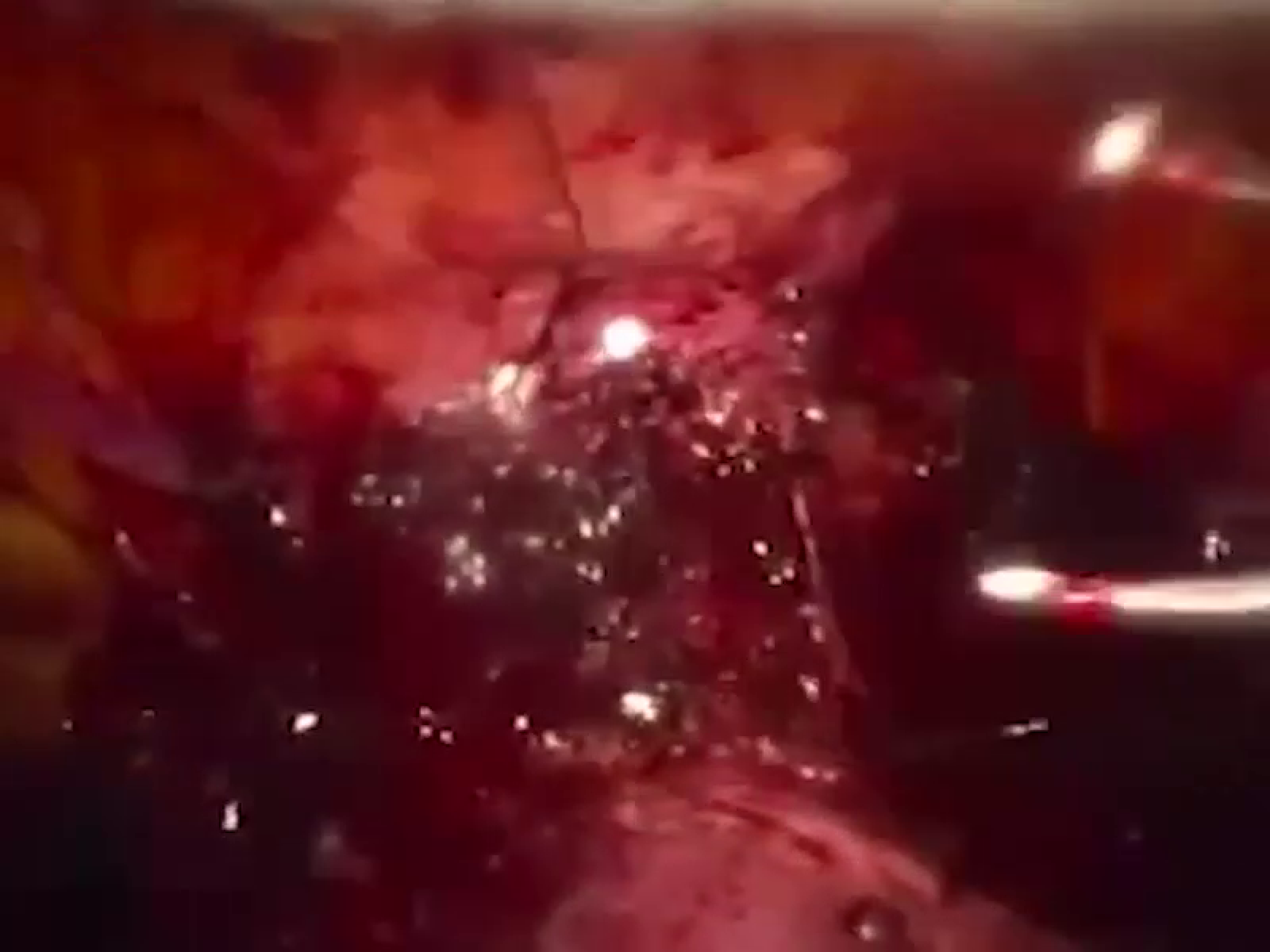

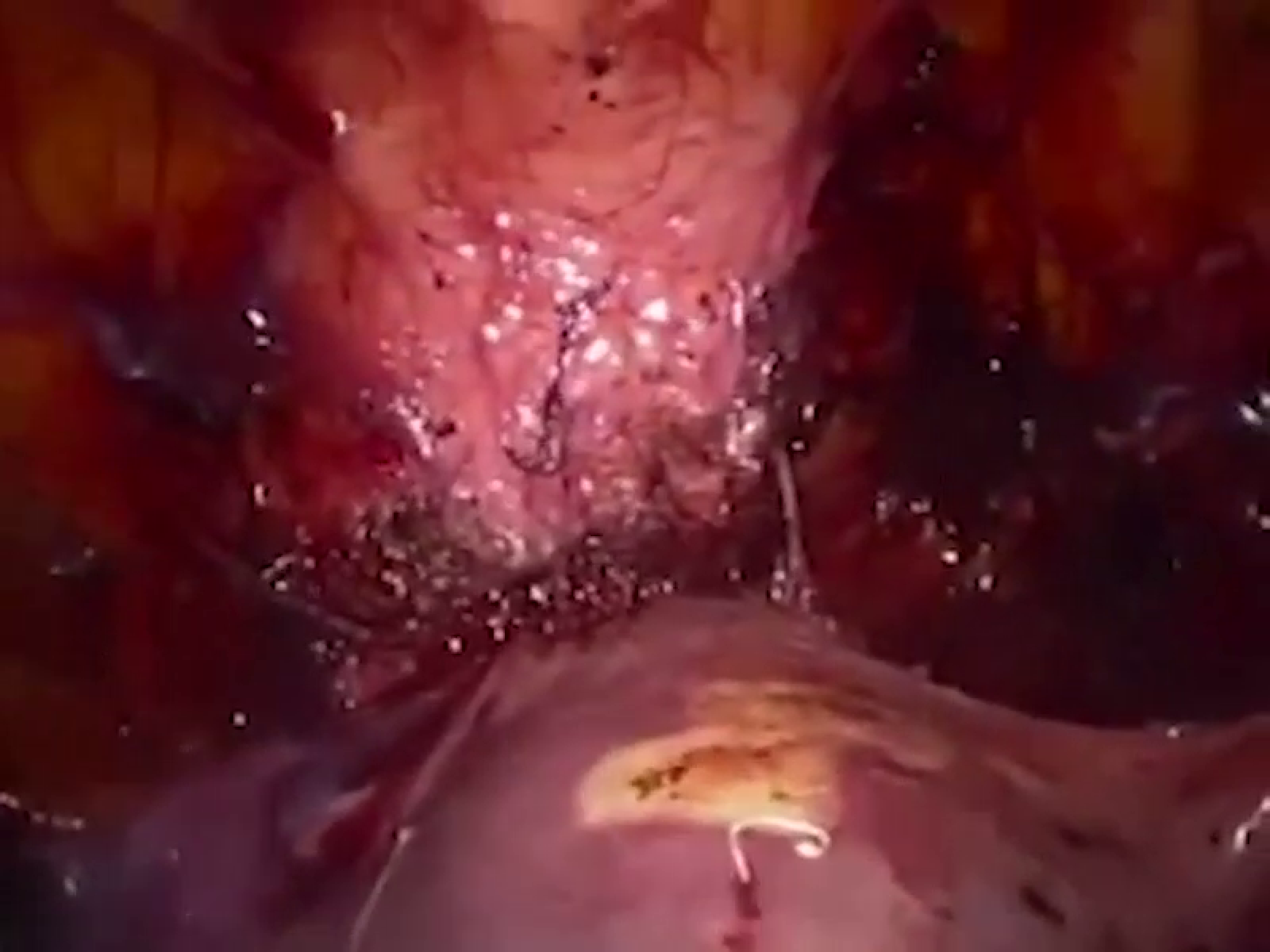

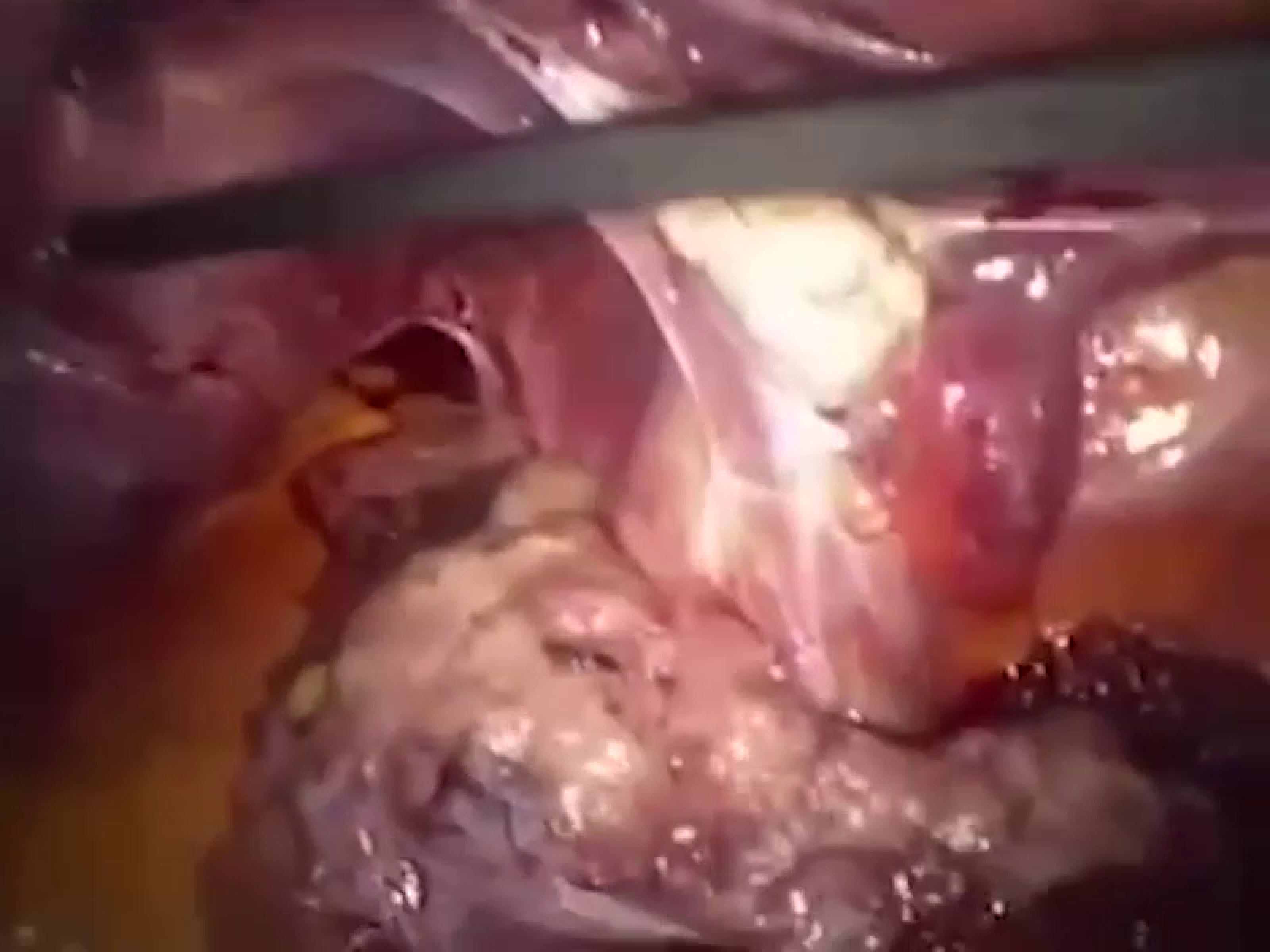

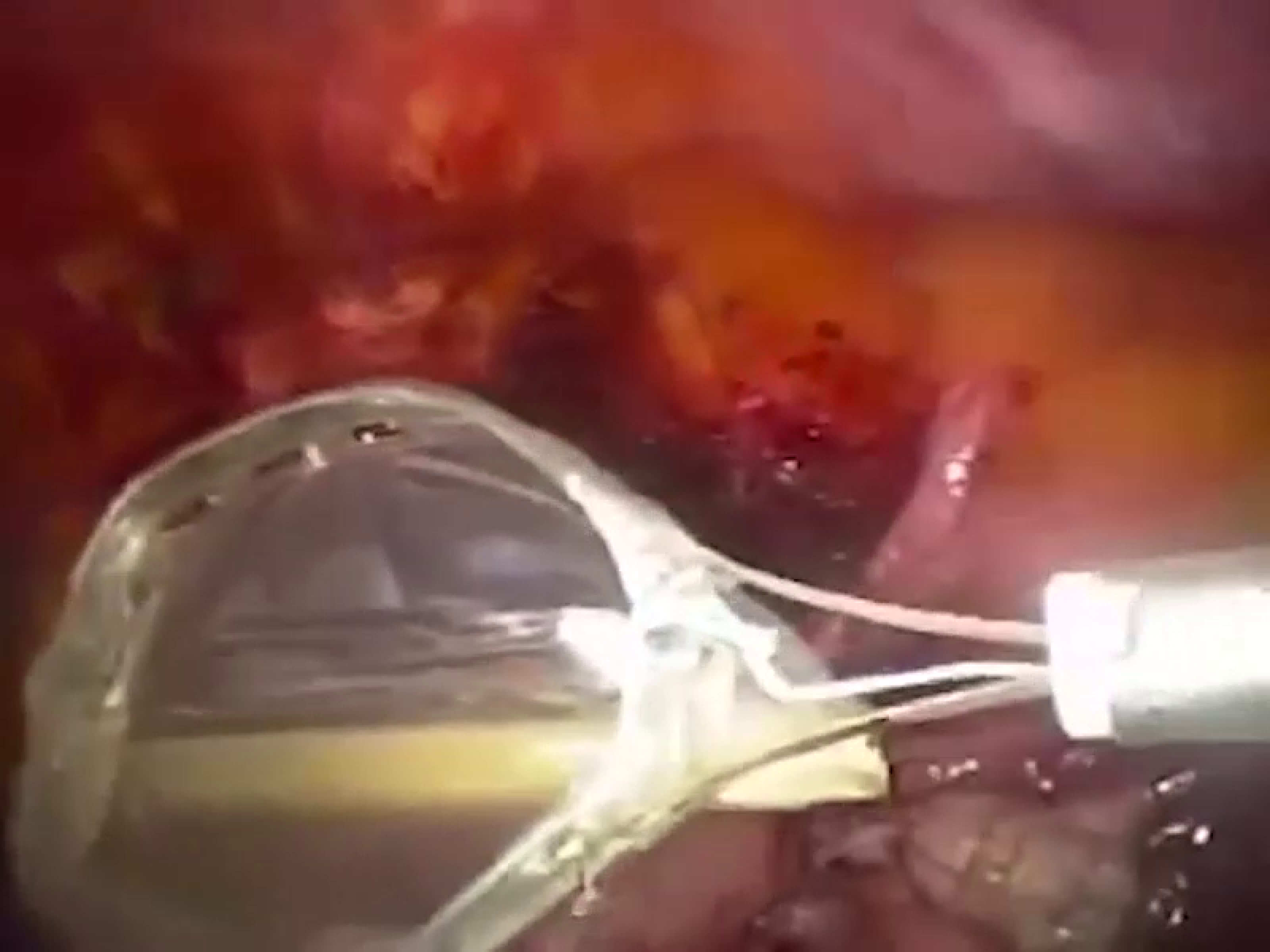

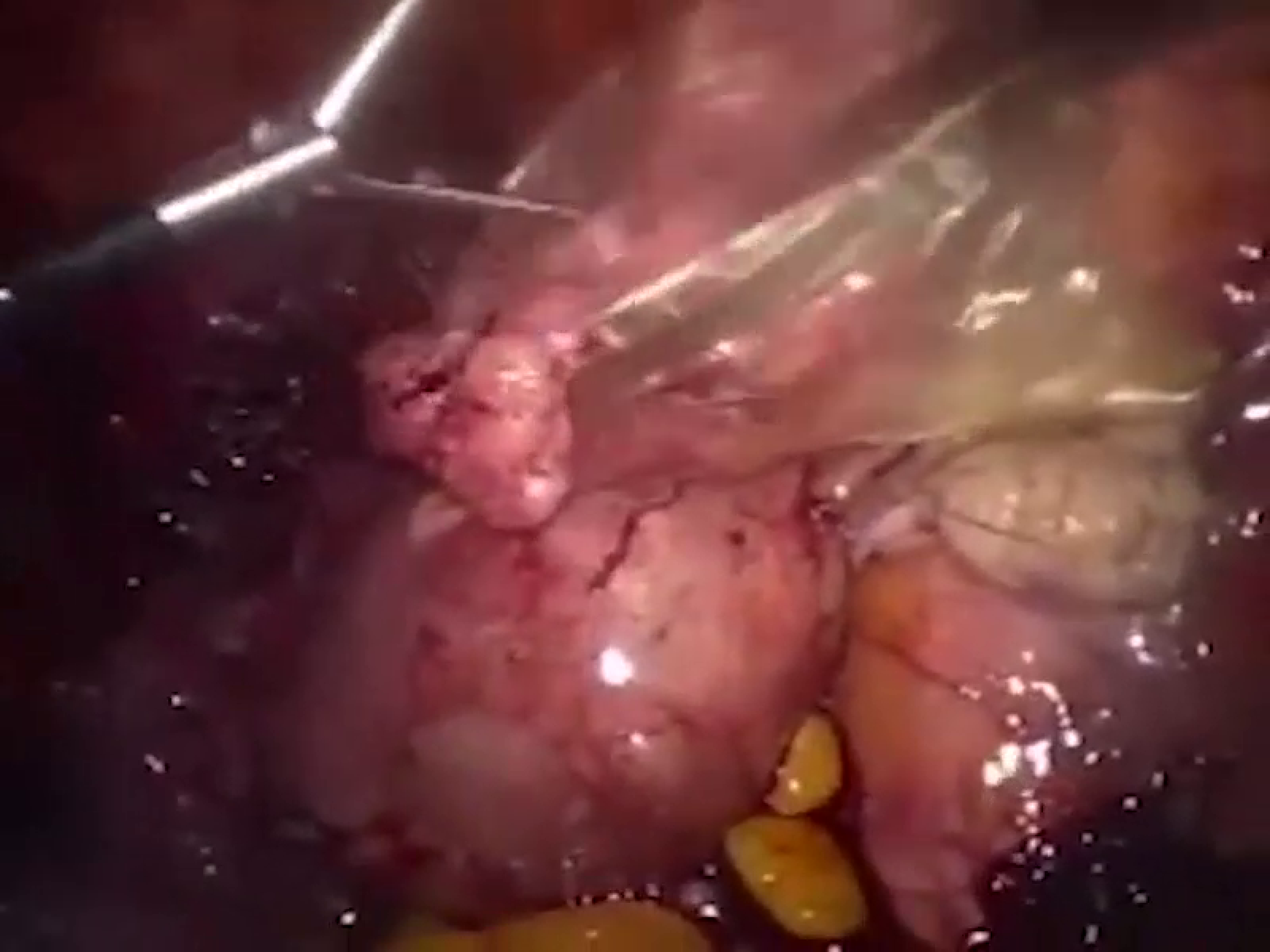

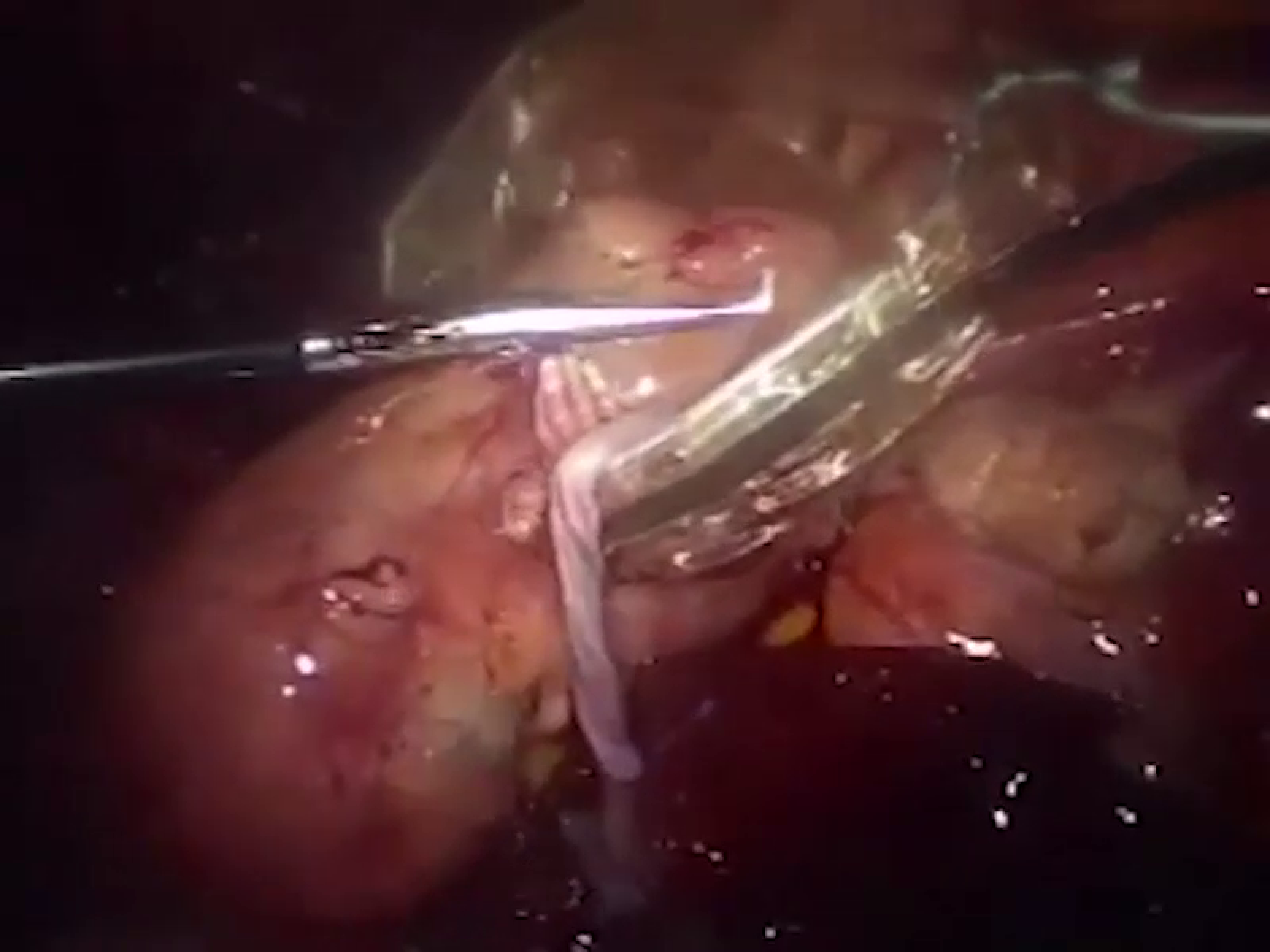

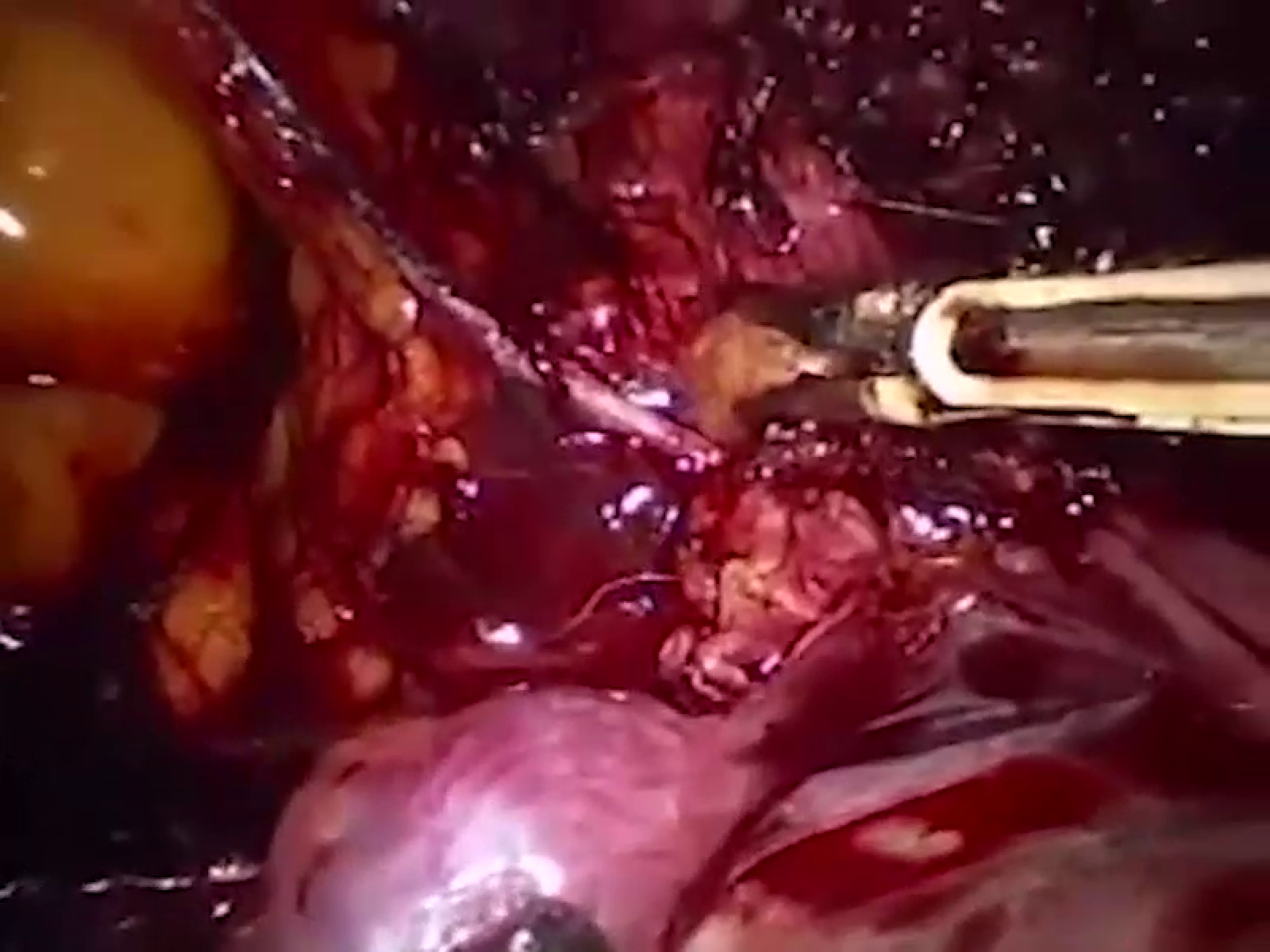

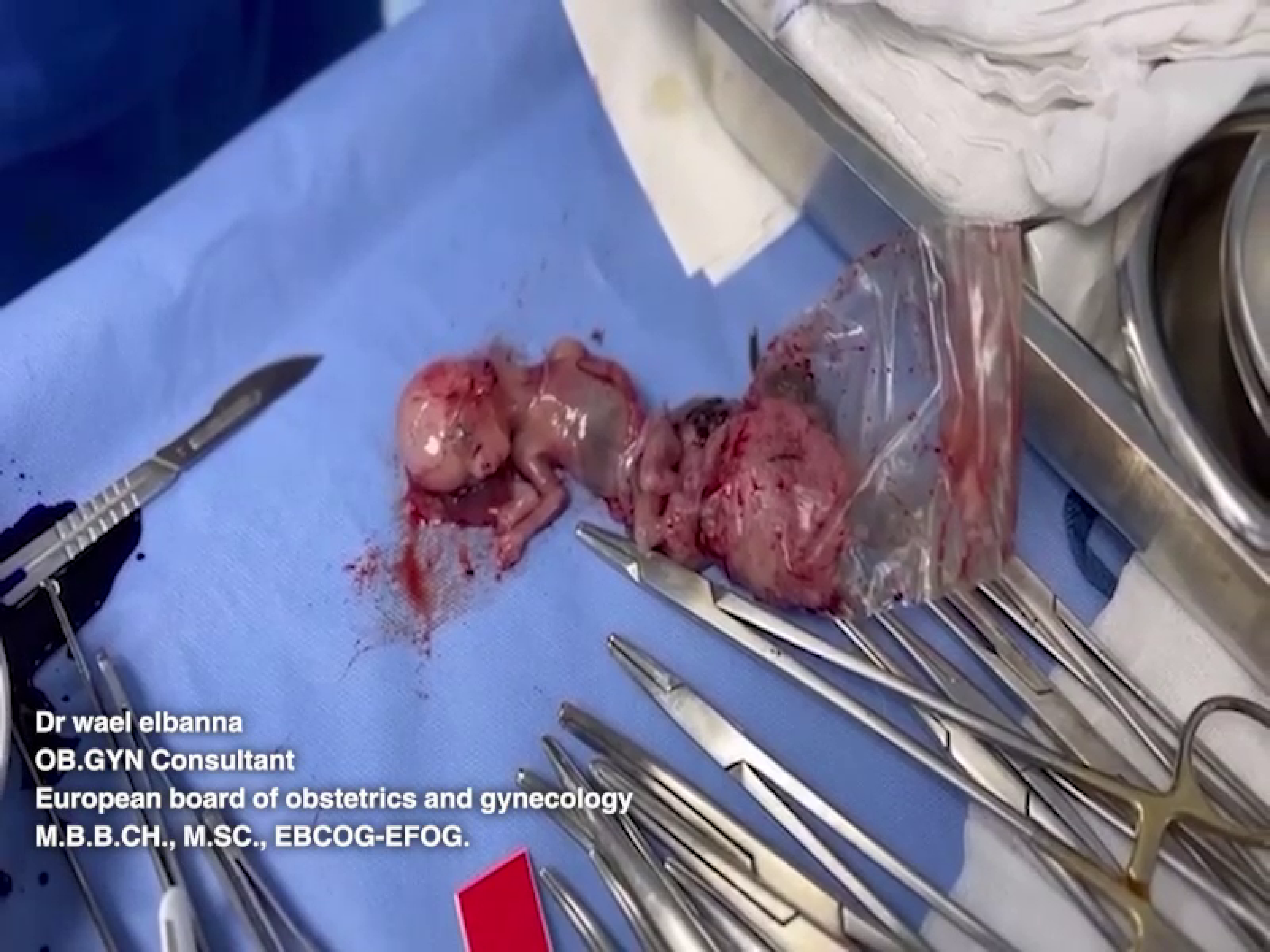
Dr wael elbanna
OB.GYN Consultant
European board of obstetrics and gynecology
M.B.B.CH., M.SC., EBCOG-EFOG.
